# Declining centromere strength compromises meiotic chromosome segregation in aging oocytes

**DOI:** 10.1083/jcb.202606132

**Published:** 2026-10-19

**Authors:** Tom Lemonnier, Marie Dumont, Jiyeon Leem, Aysia Khan, Hossein Yazdkhasti, Vitaly Zimyanin, Ani Khutsaidze, Stefanie Redemann, Daniele Fachinetti, Binyam Mogessie

**Affiliations:** 1 Department of Molecular, Cellular and Developmental Biology, Yale University, New Haven, CT, USA; 2 Department of Obstetrics, Gynecology and Reproductive Sciences, Yale University School of Medicine, New Haven, CT, USA; 3 Yale Center for Research on Aging, Yale School of Medicine, New Haven, CT, USA; 4 Institut Curie, PSL Research University, Sorbonne Université, CNRS UMR144 & 3664, Paris, France; 5 Department of Molecular Physiology and Biological Physics, University of Virginia School of Medicine, Charlottesville, VA, USA; 6 Center for Membrane and Cell Physiology, University of Virginia School of Medicine, Charlottesville, VA, USA; 7 Department of Cell Biology, University of Virginia School of Medicine, Charlottesville, VA, USA

## Abstract

Maternal age is the primary risk factor for aneuploidy in mammalian oocytes, but the molecular mechanisms that preserve meiotic chromosome segregation fidelity during aging remain incompletely understood. Using ultrastructural analysis, we observe that spindle microtubules in aged mouse oocytes tend to exhibit increased misorientation and shorter interaction lengths, whereas microtubule number and density remain largely unchanged. We demonstrate that the levels of the key centromeric components CENP-A and CENP-C are reduced in oocytes from aged mice. Acute degradation of CENP-A during meiosis I affects CENP-C stability at centromeres and causes chromosome segregation errors in young oocytes, a phenotype not observed in mitosis. The expression of exogenous CENP-C rescues these defects via CENP-B–dependent centromere binding, supporting a link between centromere weakening and meiotic errors. This intervention also partially improves chromosome alignment in oocytes from older animals. Together, these findings implicate centromere weakening, alongside cytoskeletal disorganization, in the age-related decline in chromosome segregation fidelity in oocytes.

## Teaser

Lemonnier, Dumont, and colleagues show that reproductive aging is associated with weakened centromere function and altered spindle microtubule organization in mammalian oocytes. Their findings support a role for reduced CENP-A–dependent centromere function in chromosome segregation errors and highlight multiple vulnerabilities that may compromise egg quality with advancing maternal age.

## Introduction

Accurate chromosome segregation during meiosis is essential for reproductive success and the formation of healthy embryos. Errors in chromosome segregation during female meiosis are widespread and lead to aneuploidy (a condition with abnormal chromosome numbers), which typically results in embryo deaths and accounts for nearly 35% of miscarriages (Herbert et al., 2015). Fewer than 5% of chromosomal abnormalities detected in human pregnancies arise from errors in male meiosis (Hassold and Hunt, 2001; Herbert et al., 2015). When compatible with life, embryo aneuploidy frequently leads to genetic conditions such as Down’s syndrome and Turner syndrome, with respective incidences of about 1 in 1,000 and 1 in 2,000 live births worldwide. In humans and other mammals, chromosome segregation fidelity declines sharply with age, leading to increased oocyte aneuploidy and a higher risk of infertility, miscarriage, and developmental disorders (Hassold and Hunt, 2001; Herbert et al., 2015; Nagaoka et al., 2012). Age-related loss of sister chromatid cohesion is a well-established driver of these errors (Chiang et al., 2010; Chiang et al., 2011; Lister et al., 2010; Liu and Keefe, 2008; Mihalas et al., 2024). However, reproductive aging is multifactorial (Secomandi et al., 2022), and additional contributing mechanisms likely exist yet remain poorly understood.

The centromere plays a central role in chromosome segregation by assembling the kinetochore, a multiprotein complex that mediates microtubule attachment and spindle checkpoint signaling in both meiosis and mitosis (McKinley and Cheeseman, 2016; Mellone and Fachinetti, 2021; Monda and Cheeseman, 2018; Musacchio and Salmon, 2007; Salinas-Luypaert and Fachinetti, 2024). Centromeres are assembled on long stretches of repetitive DNA bound by the centromeric protein B (CENP-B) in a DNA sequence–dependent manner (Yoda et al., 1992). In addition to unique DNA sequences, centromeres have a specialized chromatin domain enriched with the histone H3 variant CENP-A, which serves as the epigenetic mark for centromere position (McKinley and Cheeseman, 2016; Mellone and Fachinetti, 2021; Monda and Cheeseman, 2018; Salinas-Luypaert and Fachinetti, 2024). Here, CENP-A lays the foundation for the hierarchical assembly of the inner kinetochore, beginning with the recruitment of CENP-C (Fachinetti et al., 2013) and the assembly of the constitutive centromere-associated network (CCAN) (Cheeseman and Desai, 2008; Monda and Cheeseman, 2018). The outer kinetochore, composed of the KMN network (KNL1, MIS12, NDC80), builds on this inner scaffold to form robust microtubule attachments (Cheeseman and Desai, 2008; Monda and Cheeseman, 2018). The CENP-A–dependent epigenetic memory of centromere identity is conserved in mammalian meiosis, where it also defines sites for kinetochore assembly (Das et al., 2020). This is particularly important because mammalian oocytes are arrested in prophase I for extended periods. In mouse oocytes, CENP-A nucleosomes loaded before prophase I arrest are sufficient to sustain centromere function throughout the female reproductive lifespan (Smoak et al., 2016), even in the absence of de novo assembly (Das et al., 2023).

Centromere strength, defined by centromere size and the levels of centromeric and kinetochore proteins that determine its ability to bind microtubules, is suggested to be a crucial factor in ensuring accurate chromosome segregation (Lampson and Black, 2017). In mitotic cells, chromosomes with lower levels of centromeric proteins are more susceptible to segregation errors than those with stronger centromeric features (Dumont et al., 2020). In mammalian meiosis, differences in centromere strength can bias chromosome segregation outcomes through meiotic drive (Henikoff et al., 2001). In mice, centromeres are composed of arrays of minor satellite DNA repeats that form the CENP-A–containing chromatin domain (Guenatri et al., 2004). Homologous chromosomes with centromeres containing more minor satellite repeats assemble more CENP-A and recruit higher levels of pericentromeric microtubule-destabilizing activity, which biases their segregation toward the egg rather than the polar body (Akera et al., 2019; Chmátal et al., 2017; Iwata-Otsubo et al., 2017). These observations suggest that a decline in centromere strength could contribute to chromosome segregation errors and aneuploidy in reproductive aging.

Previous studies have shown that centromeric CENP-A levels decline with reproductive age in mouse oocytes and that aging is accompanied by centromeric chromatin decompaction, kinetochore fragmentation, and an increased risk of incorrect kinetochore–microtubule attachments (Smoak et al., 2016; Yin et al., 2023; Zielinska et al., 2019). However, the functional importance of this CENP-A decline remains uncertain because acute CENP-A depletion after DNA replication does not impair chromosome segregation during the first mitotic division following depletion (Hoffmann et al., 2016). This dispensability raises a central question: does age-associated CENP-A decline have functional consequences during female meiosis, or can meiotic centromeres support chromosome segregation without a preexisting CENP-A pool, such as during mitosis? Resolving this question requires directly depleting CENP-A during meiotic maturation and determining whether the resulting reduction in centromere strength compromises kinetochore function and chromosome segregation.

While centromeres and kinetochores define the sites of microtubule attachment, the fidelity of chromosome segregation also depends on the structural integrity of the meiotic spindle (Mogessie et al., 2018). Spindle architecture arises from the coordinated actions of motor proteins and nonmotor microtubule–associated proteins (MAPs) that organize and stabilize microtubule arrays (Subramanian et al., 2010; Walczak and Heald, 2008). Reproductive aging disrupts actin- and microtubule-dependent mechanisms that regulate chromosome segregation in oocytes, indicating broad cytoskeletal disorganization that could impair segregation accuracy (Dunkley and Mogessie, 2023; Mogessie and Schuh, 2017; Nakagawa and FitzHarris, 2017). Specifically, how spindle architecture is perturbed during aging and drives meiotic errors remains unresolved.

In this study, we examined the roles of CENP-A and centromere strength in accurate chromosome segregation in oocytes from reproductively young and older females. Using 3D electron tomography, quantitative live-cell imaging, targeted protein degradation via TRIM-Away, and centromere-specific fluorescence in situ hybridization (FISH) probes, we show that acute centromere weakening compromises meiotic chromosome segregation in mouse oocytes. Furthermore, our results suggest that spindle disorganization may represent an additional, age-associated vulnerability contributing to oocyte aneuploidy.

## Results

### Ultrastructural analysis suggests altered microtubule alignment and interactions within spindles of aged oocytes

**Figure S1.**
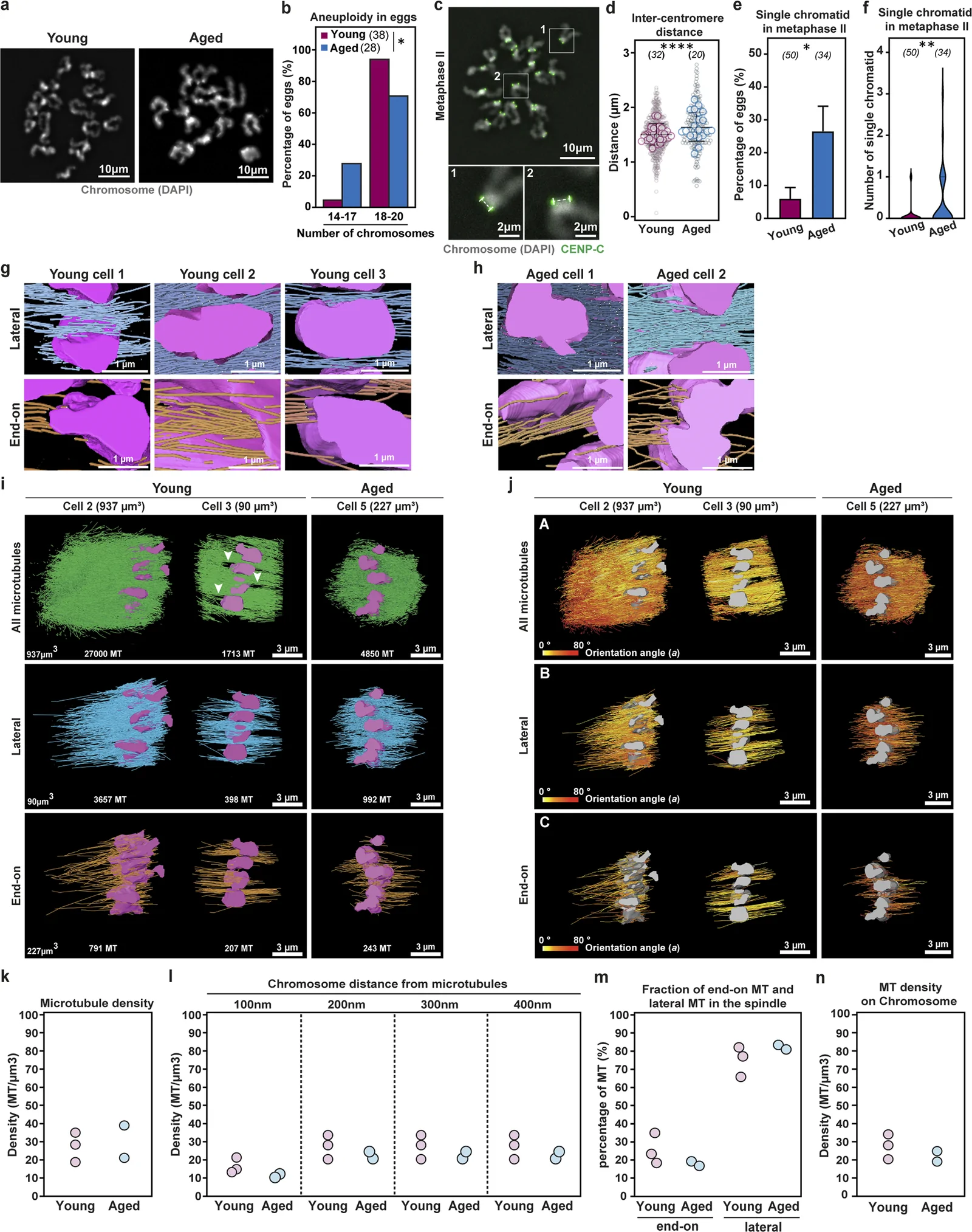
**Ultrastructural and quantitative analyses of metaphase II spindles in young and aged eggs. (a)** Representative maximum-intensity projections (DAPI) of metaphase II chromosome spreads from young and aged eggs. **(b)** Distribution of chromosome numbers per egg in young (red) and aged (blue) C57BL/6 mice. The number of oocytes analyzed is indicated in brackets. Statistical significance was assessed using a chi-square test, *P < 0.03. **(c)** Representative maximum-intensity projections of immunofluorescence images showing CENP-C (green) and chromosomes (gray). Insets highlight magnified regions. **(d)** Intercentromere distance measured between paired CENP-C signals on individual metaphase II chromosomes, as illustrated in c. Each dot represents the mean value per egg. A total of 534 and 222 kinetochores were analyzed in the young and aged groups, respectively. The number of eggs analyzed per condition is indicated in brackets. Mann–Whitney test, ****P < 0.0001. **(e)** Percentage of eggs containing single chromatids in young (red) and aged (blue) C57BL/6 mice. The number of eggs analyzed per condition is indicated in brackets. Fisher’s exact test, *P = 0.011. **(f)** Distribution of single chromatids per egg shown as violin plots for young (red) and aged (blue) C57BL/6 mice. The number of eggs analyzed per condition is indicated in brackets. Mann–Whitney test, **P = 0.007. **(g and h)** Higher magnification views of 3D tomographic reconstructions show lateral and end-on microtubule–chromosome interactions in young and aged eggs. Representative reconstructions from three young (Young cells 1–3; g) and two aged (Aged cells 1–2; h) mouse eggs are shown. In each group, the top row depicts lateral microtubule–chromosome interactions, with laterally associated microtubules rendered in cyan and chromosomes in magenta. The bottom row shows end-on microtubule–chromosome interactions from the corresponding cells, with end-on microtubules rendered in orange and chromosomes in magenta. Scale bars, 1 µm. **(i)** Partial 3D tomographic reconstructions of metaphase II spindles from representative young (cells 2 and 3) and aged (cell 5) eggs. All microtubules are shown in green (top), lateral interactions in blue (middle), and end-on attachments in orange (bottom). Chromosomes are shown in magenta. Arrowheads indicate regions lacking microtubules in front of chromosomes. Reconstruction volume and total microtubule (MT) number are indicated. **(j)** Orientation angle analysis of microtubules from the reconstructions in i. Microtubules are color-coded by their angle relative to the spindle long axis, with yellow indicating 0° and red indicating 80°. Analyses are shown for all microtubules (A, top), lateral interactions (B, middle), and end-on attachments (C, bottom). Chromosomes are shown in gray. **(k)** Microtubule density within spindles from young (pink) and aged (blue) eggs. (**l)** Microtubule density as a function of distance from chromosomes, measured at 100, 200, 300, and 400 nm in young (pink) and aged (blue) eggs. **(m)** Fraction of end-on and lateral microtubule interactions within spindles from young (pink) and aged (blue) eggs. **(n)** Microtubule density per chromosome in young (pink) and aged (blue) eggs.

**Figure 1.**
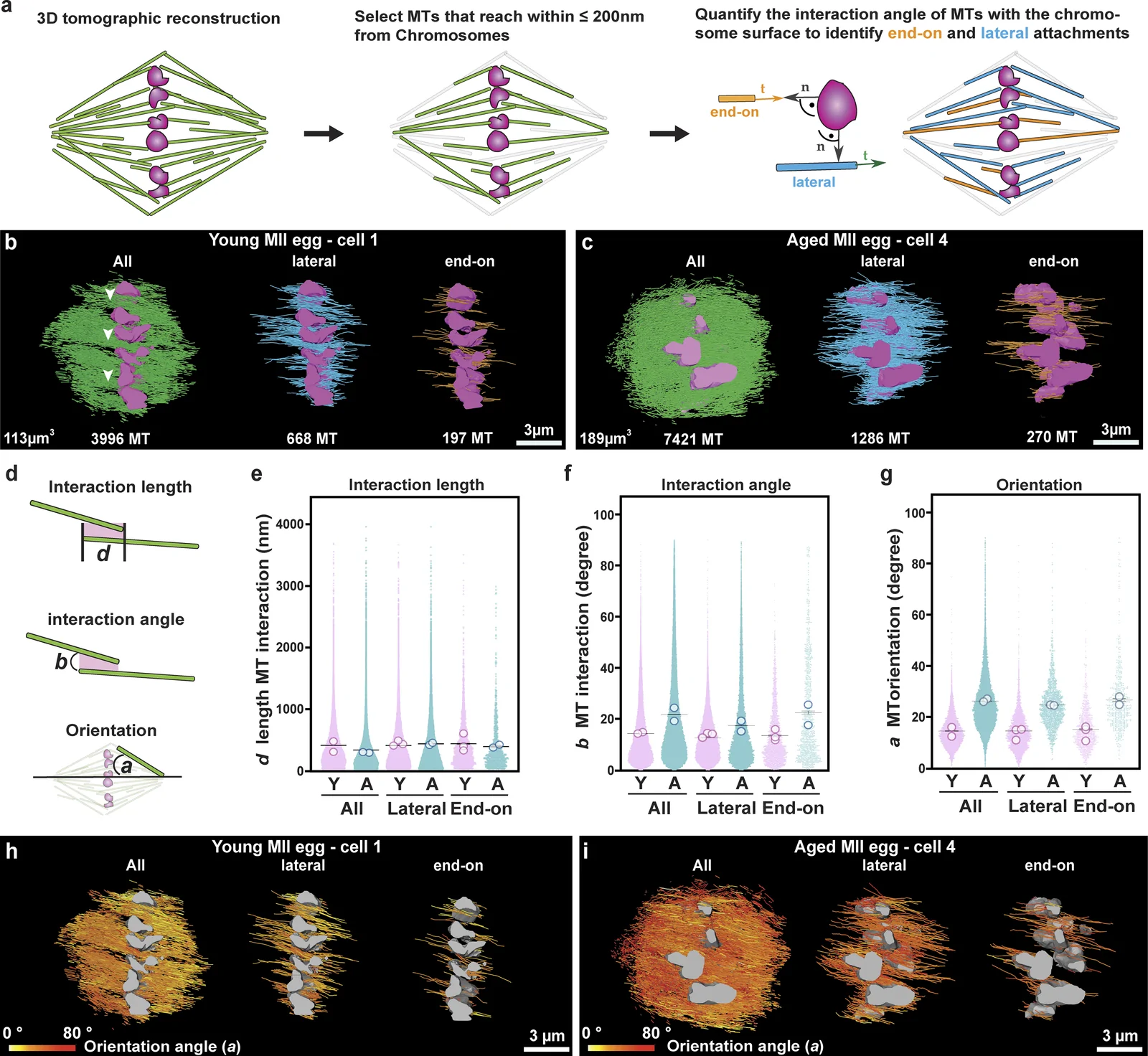
**Ultrastructural analysis identifies age-associated differences in microtubule organization in mammalian eggs. (a)** Cartoon illustrating microtubule classification. Microtubules (green) that reach within 200 nm of the chromosome surfaces are selected as potentially interacting with chromosomes (magenta). Subsequently, microtubules are separated into those that interact laterally (blue) and those that interact end-on (orange) with the chromosome surface. **(b and c)** Representative partial 3D tomographic reconstruction of metaphase-stage meiosis II spindles of a young (b) and an aged (c) egg. All microtubules in the reconstruction are shown in green (left), microtubules that interact laterally are shown in blue (middle), and end-on interacting microtubules are shown in orange (right). Chromosomes are shown in magenta. The number of microtubules and the reconstruction volume are indicated. Lateral and end-on microtubules correspond to microtubules extending within 200 nm of chromosome surfaces. **(d)** Cartoons depicting quantifications, including definitions of interaction length (d), interaction angle (b), and microtubule orientation along the spindle axis (a). **(e–g)** Plot of interaction length (e), interaction angle for each interaction (f), and orientation angle along the spindle axis (g) for all microtubules (left), lateral microtubules (middle), and end-on microtubules (right) in young (pink) and aged (blue) spindles. Large open circles indicate mean values for individual spindle reconstructions. Data were obtained from three young and two aged eggs. Given the limited number of independent biological samples, no inferential statistical comparison between age groups was conducted. **(h and i)** Representative images showing orientation angles of all (left), laterally (middle), and end-on (right) attached microtubules in young (h) and aged (i) eggs, corresponding to b and c. Microtubules are color-coded by their orientation relative to the long spindle axis, with yellow indicating 0° and red 80°. Chromosomes are shown in gray.

Accurate chromosome segregation depends on spindle structural integrity, which raises the possibility that aneuploidy in aged oocytes may arise from disrupted spindle architecture. Consistent with this prediction, microtubule dynamics are aberrant in aged oocytes (Nakagawa and FitzHarris, 2017), and age-related spindle actin disruption synergizes with cohesion loss to accelerate the onset of aneuploidy (Dunkley and Mogessie, 2023; Leem et al., 2025). In addition, defects in mitotic spindle architecture, including altered microtubule bundling or orientation, could impair chromosome movements independently of kinetochore–microtubule attachment number (Matković et al., 2022; Tolić, 2018). To determine whether meiotic spindle disorganization represents a distinct age-associated vulnerability, we performed ultrastructural analysis of spindles from metaphase II–stage mouse eggs. We first confirmed that eggs obtained by *in vitro* maturation of oocytes from reproductively older (50-wk-old) females exhibited a higher incidence of chromosome loss than those from reproductively younger (8–12-wk-old) females (Fig. S1, a and b), a hallmark of oocyte aging (Herbert et al., 2015). Consistent with cohesion depletion, aged eggs showed increased intercentromere distances (Fig. S1, c and d) and a higher frequency of premature sister chromatid separation (Fig. S1, e and f) (Hassold and Hunt, 2001; Herbert et al., 2015; Nagaoka et al., 2012). We then performed serial-section 3D electron tomography to analyze spindle microtubule organization and identify structural defects related to reproductive aging (Lindow et al., 2018; Lindow et al., 2021; Redemann et al., 2014) (Fig. 1 a). We generated five partial reconstructions of meiotic spindles from CD1 outbred mouse eggs: three from young eggs (cells 1, 2, and 3) and two from aged eggs (cells 4 and 5) (Fig. 1, b and c; Fig. S1, g–j; and Videos 1 and 2). Microtubule density varied across the five reconstructed cells, with no consistent difference between the three young and the two aged eggs (1: 35 MT/µm^3^, 2: 29 MT/µm^3^, 3: 19 MT/µm^3^, 4: 39 MT/µm^3^, 5: 21 MT/µm^3^) (Fig. S1 k), in contrast to prior fluorescence microscopy–based measurements (Yin et al., 2023). We next analyzed microtubules within 200 nm of chromosome surfaces to identify those likely to interact with chromosomes and kinetochores. We selected this distance threshold based on previously established parameters for quantifying chromosome–microtubule interactions in *Caenorhabditis elegans* and mammalian systems (Kiewisz et al., 2022; Redemann et al., 2017). Changing the distance threshold from 100 to 400 nm did not suggest differences in chromosome–microtubule interactions in young and aged eggs (Fig. S1 l). Across all reconstructions, we found that 15–35% of microtubules in each dataset extended within 200 nm of chromosome surfaces (1: 22%, 2: 16%, 3: 35%, 4: 21%, 5: 25%) (Fig. 1 b). We therefore focused on this population of microtubules and further examined their spatial organization relative to chromosomes. To distinguish end-on kinetochore–microtubule attachments from lateral interactions, we quantified chromosome–microtubule interaction angles by measuring the angle formed between the microtubule tangent (*t*) and a line perpendicular to the nearest point on the chromosome surface (*n*) (Fig. 1 a), following a previously described method (Redemann et al., 2018). This angle-based criterion is purely geometric because it classifies attachment orientation relative to the local chromosome surface, independent of centromere architecture. Across the five reconstructions, the proportions of end-on (1: 23%, 2: 18%, 3: 34%, 4: 17%, 5: 20%) and lateral (1: 77%, 2: 82%, 3: 66%, 4: 83%, 5: 80%) kinetochore–microtubule interactions showed no consistent age-related pattern (Fig. S1 m). Normalization of end-on interactions to chromosome volume likewise showed no consistent pattern between the three young and two aged eggs (1: 28 MT/µm^3^, 2: 34 MT/µm^3^, 3: 20 MT/µm^3^, 4: 26 MT/µm^3^, 5: 18 MT/µm^3^) (Fig. S1 n). Therefore, within this limited set of reconstructions, we did not observe a consistent age-related change in the number or orientation of chromosome–microtubule interactions.

**Video 1.** **3D tomographic reconstruction of cell 1.** Movie of the 3D tomographic reconstruction of the young MII egg 1. Microtubules are shown in green, chromosomes in magenta.

**Video 2.** **3D tomographic reconstruction of cell 4.** Movie of the 3D tomographic reconstruction of the aged MII egg 4. Microtubules are shown in green, chromosomes in magenta.

Based on these results, we hypothesized that chromosome segregation errors in aged oocytes may arise from altered interfilament interactions within microtubules. We tested this by analyzing interactions between individual microtubules within reconstructed spindle volumes and within the subset of microtubules that interact with chromosomes in end-on or lateral configurations. Using parameters from our previous spindle reconstruction studies (Redemann et al., 2017), we defined microtubules within 100 nm of each other as potentially interacting and measured the length and angle of these interactions (Fig. 1 d). Interaction lengths, the length over which microtubules maintained a distance of ≤100 nm, were reduced in aged eggs. Across the reconstructed cells, mean interaction length was lower in aged eggs than in young eggs (376 ± 1.1 nm versus 412 ± 1.9 nm, SEM; Fig. 1 e). Mean interaction length of end-on attaching microtubules was also lower in aged eggs than in young eggs (394 ± 13 nm versus 440 ± 7.9 nm, SEM; Fig. 1 e). In contrast, lateral interactions between microtubules and chromosomes showed a modest increase in length in aged eggs (438 ± 2.9 nm, SEM) compared with young eggs (409 ± 2.0 nm, SEM) (Fig. 1 e). Consistent with these changes in interaction length, microtubule interactions in aged eggs also occurred at higher angles. Across all interactions, the average angle increased from 14° ± 0.06° (SEM) in young eggs to 22° ± 0.06° (SEM) in aged eggs (Fig. 1 f). This shift was also evident in both subsets, with the end-on category of microtubule interactions increasing from 13° ± 0.2°–22° ± 0.66°, and lateral interactions increasing from 13° ± 0.05°–17° ± 0.1° (Fig. 1 f). These results suggest that meiotic spindle microtubules in aged eggs are less well organized, consistent with defects in microtubule alignment and interaction. We investigated this further by analyzing the orientation of microtubules relative to the long axis of the metaphase II spindle (Fig. 1 d). Mean microtubule orientation angles were higher in the aged reconstructions across all categories (Fig. 1 g). For all microtubules, the average angle increased from 15° ± 0.08° (SEM) in young eggs to 26° ± 0.1° (SEM) in aged eggs. This increase was also observed for both end-on (15° ± 0.2°–27° ± 0.6°) and lateral attachments of microtubules (15° ± 0.12°–25° ± 0.21°) (Fig. 1, g–i).

Across this limited set of reconstructions, microtubule interaction lengths were shorter, and interaction and orientation angles were larger in the two aged eggs than in the three young eggs, with no apparent reduction in the number of chromosome-associated microtubules. Although the limited number of independent samples means these observations require validation in more oocytes, these structural differences could affect coordinated force generation by spindle microtubules and underlie chromosome segregation errors in aged eggs.

### Centromere composition is uniformly altered across all chromosomes in oocytes from reproductively older females

**Figure 2.**
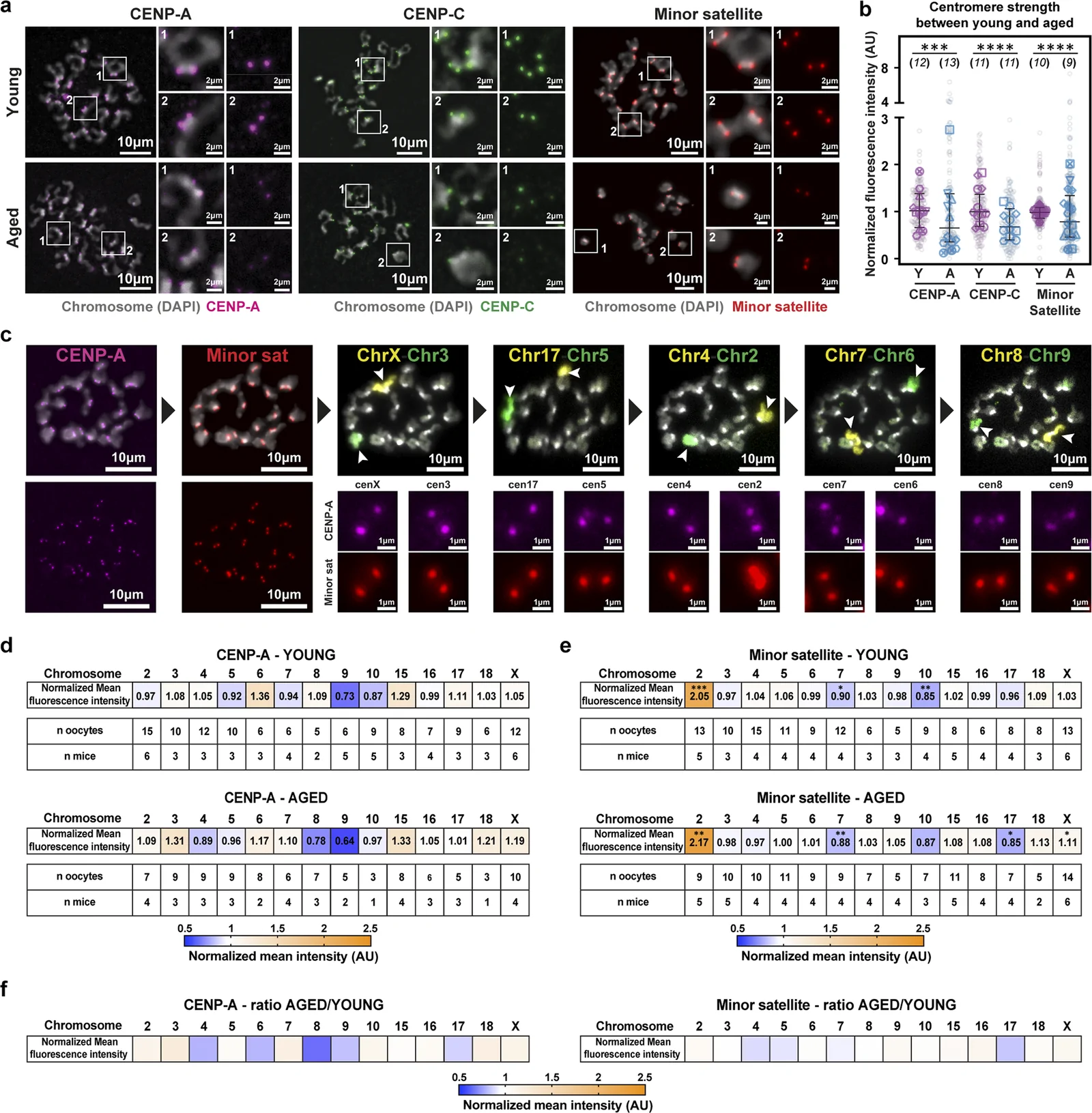
**Maternal aging reduces centromeric CENP-A and CENP-C levels and alters satellite DNA signals in mouse eggs. (a)** Representative maximum-intensity projection immunofluorescence images of CENP-A (magenta), CENP-C (green), and minor satellite FISH signals (red), merged with DAPI (gray), in metaphase II chromosome spreads from eggs of young and aged C57BL/6 mice. Boxed regions indicate areas shown at higher magnification. **(b)** Normalized integrated fluorescence density of centromeric CENP-A, CENP-C, and minor satellite signals in metaphase II chromosome spreads from young (Y) and aged (A) C57BL/6 eggs. Dot plots show mean values with interquartile range for individual centromeres (*N* > 12 oocytes, *n* > 200 centromeres for CENP-A; *N* > 11 oocytes, *n* >180 centromeres for CENP-C; and *N* > 9 oocytes, *n* > 300 centromeres for minor satellite). Each small circle represents a single centromere, and each contoured circle represents the mean of a single egg derived from different mice (depicted with different shapes). Mann–Whitney test, ***P = 0.001; ****P < 0.0001. **(c)** Representative maximum-intensity projection immunofluorescence images of CENP-A immunofluorescence followed by minor satellite FISH and sequential chromosome-specific FISH (highlighted chromosomes shown in green and yellow, indicated by a white arrow) in a metaphase II chromosome spread from a young C57BL/6 egg. Panels show magnified views of identified centromeres with CENP-A and minor satellite FISH staining. **(d and e)** Heatmaps showing chromosome-specific normalized mean of CENP-A immunofluorescence (d) and minor satellite FISH fluorescence (e) in young and aged C57BL/6 mouse eggs. The normalized mean intensity value, and the number of eggs and mice analyzed are indicated for each chromosome. **(f)** Heatmaps showing the aged-to-young ratio of chromosome-specific normalized mean CENP-A immunofluorescence and minor satellite FISH signals derived from data in d and e. Statistical significance was evaluated using Wilcoxon’s signed rank test against the theoretical value of 1 (d and e). *P < 0.05, **P = 0.01, ***P < 0.001. No asterisks, P > 0.05.

**Figure S2.**
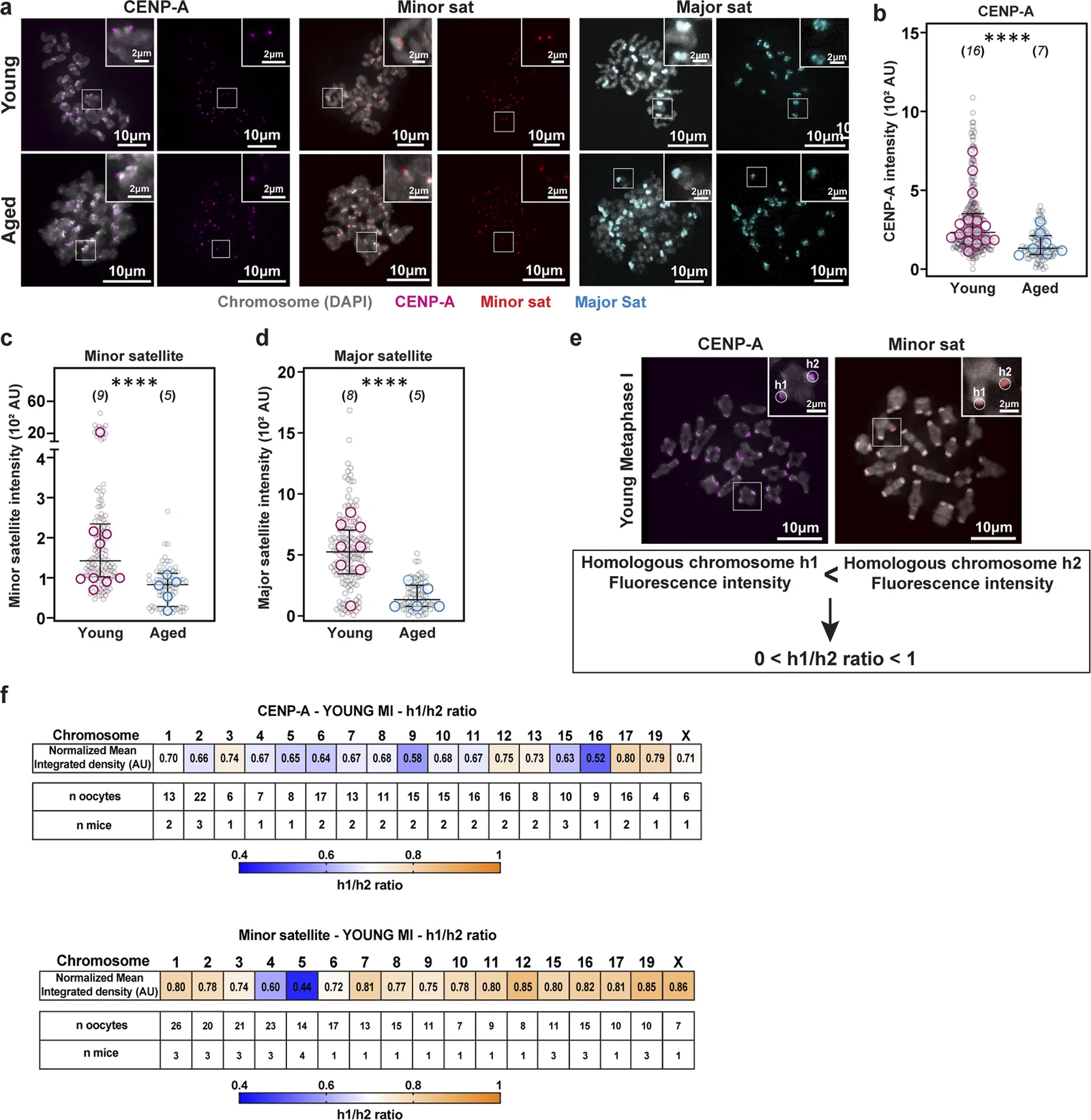
**Age-associated changes in centromere composition in oocytes and eggs. (a)** Representative maximum-intensity projections of immunofluorescence images showing CENP-A (magenta) and FISH detection of minor (red) or major (cyan) satellite sequences, merged with DAPI (gray), in metaphase II chromosome spreads from young and aged CD1 eggs. Insets show magnified regions. **(b–d)** Quantification of centromeric fluorescence intensity for CENP-A (b), minor satellite DNA (c), and major satellite DNA (d) in metaphase II chromosome spreads from young and aged CD1 eggs. Integrated density was measured within centromeric ROIs, with background subtraction from noncentromeric ROIs. Each dot represents a centromere, and contoured dots indicate the mean per egg. The number of eggs analyzed is indicated in brackets; >60 centromeres were analyzed. Data are presented as median with interquartile range. Mann–Whitney test, ****P < 0.0001. **(e)** Representative maximum-intensity projections of immunofluorescence images showing CENP-A (magenta) and minor satellite FISH (red), merged with DAPI (gray), in metaphase I chromosome spreads from young CD1 oocytes. Insets show homologous chromosome pairs (h1 and h2) used for ratio measurements. **(f)** Heatmaps showing chromosome-specific homolog intensity ratios (h1/h2) for CENP-A (top) and minor satellite DNA (bottom) in young CD1 oocytes. Ratios were calculated as the normalized mean integrated density of the weaker homolog (h1) relative to the stronger homolog (h2), as illustrated in e, where 0 < h1/h2 <1. The number of oocytes and mice analyzed per chromosome is indicated. Statistical significance was evaluated using the Wilcoxon signed rank test against the theoretical value of 1 (f). ****P < 0.0001. ROIs, regions of interest.

Centromeres serve as sites of kinetochore assembly where microtubule attachment, force transmission, and checkpoint signaling are coordinated (Cheeseman and Desai, 2008; Monda and Cheeseman, 2018; Musacchio, 2015; Musacchio and Desai, 2017; Westhorpe and Straight, 2014). Changes in the duration or turnover of kinetochore–microtubule attachments might not cause noticeable differences in attachment number or density in ultrastructural snapshots. Therefore, even when attachment numbers appear unchanged in aged eggs, centromere weakening can still impair kinetochore function by lowering the efficiency of these interactions, thereby restricting force generation and reducing chromosome segregation fidelity. We therefore investigated whether centromere integrity declines in oocytes during reproductive aging. To test this, we measured age-related changes in centromeric proteins and DNA at chromosome-specific resolution using metaphase I and II chromosome spreads. Consistent with previous observations in meiosis I (Yin et al., 2023), quantitative immunofluorescence analysis of metaphase II spreads showed lower CENP-A and CENP-C levels in aged eggs from C57BL/6 mice (Fig. 2, a and b). A similar reduction in CENP-A was observed in outbred strains (Fig. S2, a and b). We next analyzed centromeric and pericentromeric DNA. Major satellite sequences define pericentromeric chromatin and are thought to undergo structural and functional changes during aging (O'Sullivan and Karlseder, 2012; Swanson et al., 2013; Tsurumi and Li, 2012; Villeponteau, 1997). In contrast, minor satellite sequences support CENP-A deposition and kinetochore assembly (Fachinetti et al., 2013; Westhorpe and Straight, 2014), contain conserved elements necessary for chromosome segregation (Barra and Fachinetti, 2018; Chen et al., 2024; McKinley and Cheeseman, 2016; Westhorpe and Straight, 2014), and are generally expected to remain stable with age. Using FISH, we observed that signals for both minor and major satellite DNA are reduced in eggs from aged outbred mice (Fig. S2, c and d), with minor satellite signals also decreased in eggs from aged inbred mice (Fig. 2, a and b).

Since centromere strength can bias chromosome segregation outcomes during meiosis (Akera et al., 2017; Akera et al., 2019; Chmátal et al., 2017), we next asked whether age-related changes in centromere composition occur uniformly across chromosomes or preferentially affect certain chromosomes. To address this, we labeled individual chromosomes in single metaphase II–stage eggs from inbred C57BL/6 mice using immunofluorescence for CENP-A or minor satellite FISH, followed by sequential chromosome-specific FISH probes (Fig. 2 c). CENP-A fluorescence varied across chromosomes, with chromosome 9 showing the lowest normalized signal in both young and aged eggs (0.7 and 0.6, respectively; Fig. 2 d). Minor satellite signals were largely uniform across chromosomes, except for centromere 2, which showed twofold higher intensity, and centromeres 7, 10, and 17, which exhibited a modest reduction in signal (Fig. 2 e). Comparison of CENP-A and minor satellite signals between young and aged eggs for each chromosome (presented as aged/young ratio; Fig. 2 f) showed no chromosome-specific differences in the degree of decline, suggesting that centromere components are reduced uniformly across chromosomes during reproductive aging.

We next applied a similar approach to metaphase I chromosome spreads from oocytes of reproductively young outbred CD1 mice. These analyses showed heterogeneity in CENP-A levels and minor satellite sequences between centromeres of homologous chromosomes (Fig. S2, e and f). Comparing signal intensity ratios of the “weaker” centromere (lower minor satellite DNA and CENP-A) with the “stronger” homolog (higher levels of both) showed a significant difference for both CENP-A and minor satellite signal ratios (Fig. S2 f). This asymmetry was particularly evident in CENP-A for chromosomes 9 and 16 (ratios <0.6) and in minor satellite for chromosomes 4 and 5 (ratios < 0.6) (Fig. S2 f). Together, these results suggest that while centromeric components decline uniformly across chromosomes with age at the population level, notable asymmetry in centromere strength exists between homologs within individual oocytes. This suggests that intra-oocyte heterogeneity in centromeric composition may contribute to biased chromosome segregation and represent a potential mechanism underlying age-related oocyte aneuploidy.

### CENP-A is required for accurate chromosome congression, alignment, and segregation in mouse oocytes

**Figure 3.**
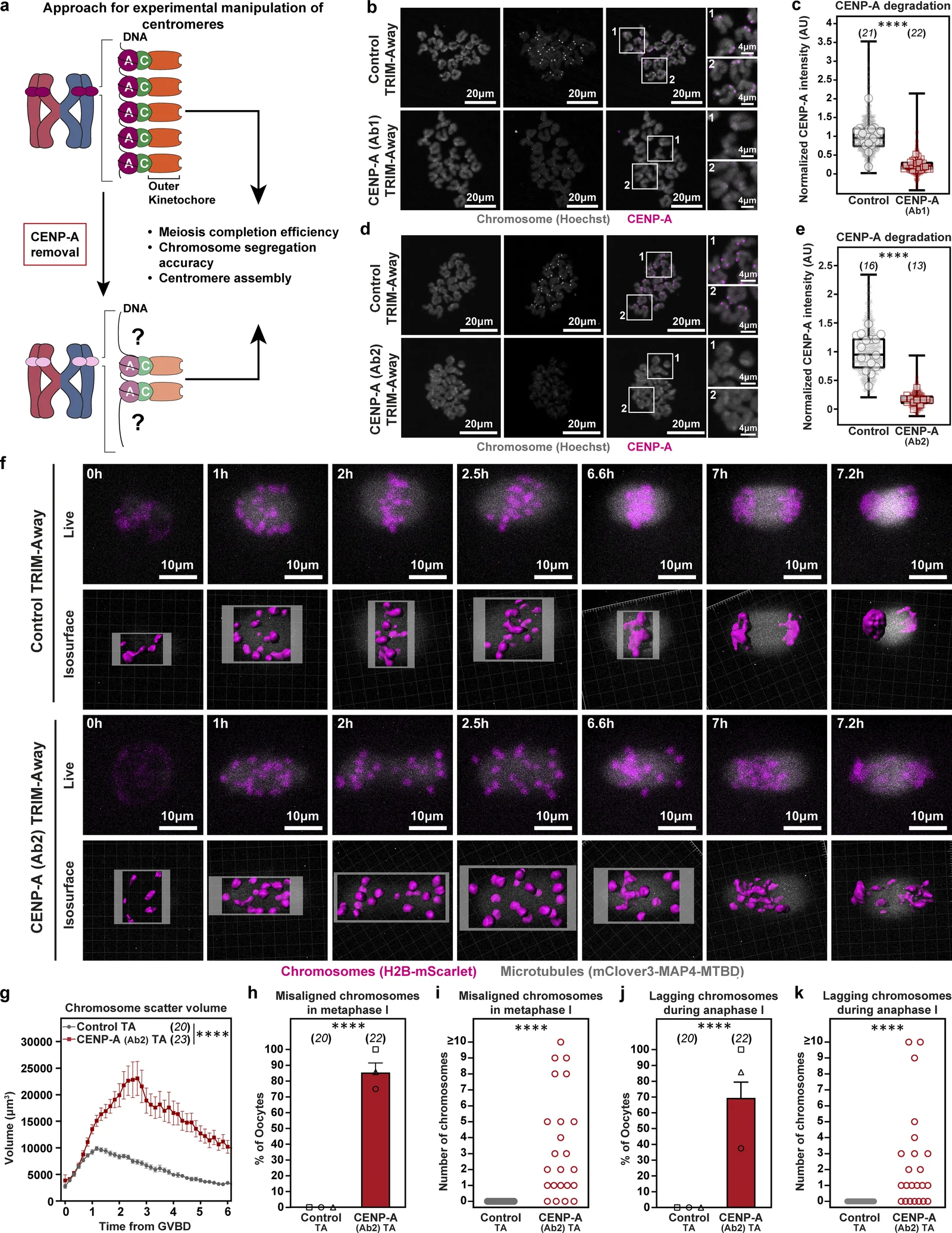
**Acute CENP-A depletion in young oocytes disrupts chromosome alignment and segregation during meiosis. (a)** Schematic illustrating the strategy for experimental manipulation of centromeres in mouse oocytes. A, CENP-A; C, CENP-C. **(b–e)** Representative maximum-intensity projection immunofluorescence images of CENP-A and chromosomes (Hoechst) in metaphase II chromosome spreads from control IgG (control) and CENP-A antibody TRIM-Away eggs (Ab1 in b, Ab2 in d), with corresponding quantification of normalized integrated fluorescence density of centromeric CENP-A signals (Ab1 in c, Ab2 in e). Boxed regions indicate areas shown at higher magnification. In the box plots, each small circle or square represents a single centromere, and each large circle or square represents the mean of a single egg. Box plots show median, interquartile range, and the minimum and maximum values. Data are from three independent experiments. ****P < 0.0001. **(f)** Representative stills from 6-min time-lapse movies starting at GVBD showing chromosomes (H2B-mScarlet) in control and CENP-A (Ab2) TRIM-Away oocytes during meiosis I (live panel). Corresponding pseudo-object–oriented bounding boxes (gray, isosurface panel) define the minimal cuboid volume enclosing all chromosomes from GVBD to the metaphase-to-anaphase transition, used as a measure of chromosome scattering. **(g)** Chromosome scatter volumes quantified from (f) over 6 h from GVBD in control and CENP-A (Ab2) TRIM-Away (TA) oocytes. Gray and red lines indicate mean values, and error bars represent the SEM. Data are from three independent experiments. ****P < 0.0001. **(h and i)** Proportion of oocytes with misaligned chromosomes (h) and number of misaligned chromosomes per oocyte (i) (identified in f) in control and CENP-A (Ab2) TRIM-Away oocytes at metaphase I (defined as the final frame before anaphase onset). In h, error bars represent the SEM and black circles indicate the mean of each independent experiment. Data are from three independent experiments. ****P < 0.0001. **(j and k)** Proportion of oocytes with severely lagging chromosomes (j) and number of lagging chromosomes per oocyte (k), identified in (f), in control and CENP-A (Ab2) TRIM-Away oocytes during anaphase I. Severely lagging chromosomes are defined as chromosomes that remain lagging 18 min after anaphase onset (three frames). In (j), error bars represent the SEM and black circles indicate the mean of each independent experiment. Data are from three independent experiments. ****P < 0.0001. Statistical significance was evaluated using two-tailed Welch’s *t* test (e and i), two-tailed Mann–Whitney test (c and k), mixed-effects analysis (g), or Fisher’s exact test (h and j). The number of oocytes analyzed per condition is indicated in brackets.

**Figure S3.**
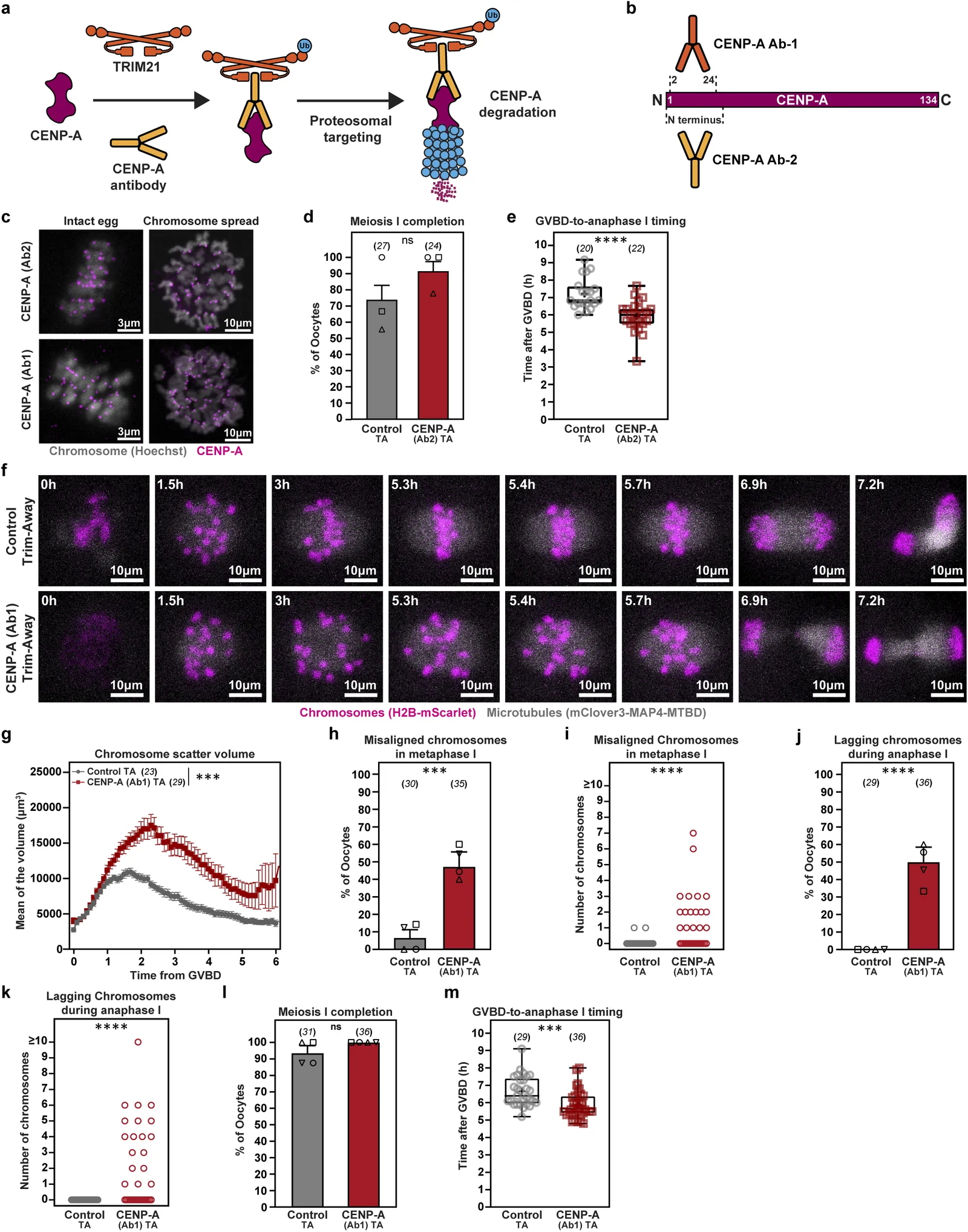
**CENP-A TRIM-Away phenotypes are reproducible using an independent custom antibody. (a)** Schematic of the TRIM-Away approach for antibody-mediated degradation of CENP-A. **(b)** Two independent antibodies were used for CENP-A degradation via TRIM-Away: a custom antibody (Ab1) recognizing a 23–amino acid epitope in the N terminus, and a commercial antibody (Ab2) recognizing an N-terminal region of CENP-A. **(c)** Representative maximum-intensity projections of immunofluorescence images showing chromosomes (Hoechst, gray) and CENP-A (magenta) in intact eggs and metaphase II chromosome spreads. CENP-A was detected using the antibodies described in b. **(d)** Proportion of oocytes completing meiosis I. Each circle represents the mean of an independent experiment. **(e)** Time from GVBD to anaphase I onset for individual oocytes. ****P < 0.0001. (**f)** Representative stills from a 6-min time-lapse imaging of chromosomes and microtubules in control and CENP-A TRIM-Away (Ab1) oocytes during meiosis I. Time is indicated relative to GVBD. **(g)** Mean chromosome scatter volume over time following GVBD in control and CENP-A TRIM-Away oocytes (Ab1). Error bars represent the SEM. Data are from four independent experiments, ***P = 0.0007. **(h and i)** Frequency of oocytes with misaligned chromosomes (h) and number of misaligned chromosomes per oocyte (i) at metaphase I, defined as the final frame prior to anaphase onset. In h, symbols represent means of independent experiments and error bars indicate the SEM. Data are from four independent experiments. (h) ***P = 0.0003. (i) ****P < 0.0001. **(j and k)** Frequency of oocytes with lagging chromosomes (j) and the number of lagging chromosomes per oocyte (k) during anaphase I. Lagging chromosomes were defined as those trailing behind the segregating chromosome mass 18 min (three frames) after anaphase onset. Data are from four independent experiments. ****P < 0.0001. **(l)** Proportion of oocytes completing meiosis I. Each dot represents the mean of an independent experiment. Data are from four independent experiments. *P = 0.2103. **(m)** Time from GVBD to anaphase I onset. Data are from four independent experiments. ***P = 0.0001. Statistical significance was evaluated using the two-tailed Mann–Whitney test (e, i, k, and m), mixed-effects analysis (g), or Fisher’s exact test (d, h, j, and l). The number of oocytes analyzed per condition is indicated in brackets.

To determine whether CENP-A loss is sufficient to cause chromosome segregation errors associated with oocyte aging, we depleted CENP-A in fully grown, prophase-arrested oocytes using the TRIM-Away method (referred to as TA in figure panels) (Clift et al., 2017) (Fig. 3 a and Fig. S3 a). This approach bypasses potential confounds arising from its deposition earlier in development (Smoak et al., 2016) and isolates its contribution to chromosome segregation during meiosis I. Although CENP-A persists throughout prophase arrest in mammalian oocytes (Smoak et al., 2016), its functional requirement during meiotic divisions remains unresolved. We microinjected prophase-arrested mouse oocytes with mRNAs encoding TRIM21 together with control IgG or one of two CENP-A antibodies (Ab1 and Ab2) to induce degradation (Fig. S3, b and c). Immunofluorescence analysis of chromosome spreads from metaphase II eggs confirmed efficient degradation of CENP-A, with protein levels reduced by 76% using Ab1 and 84% using Ab2 relative to controls (Fig. 3, b–e).

To determine how CENP-A loss affects chromosome behavior during meiosis I, we performed high-resolution live imaging of chromosomes (H2B-mScarlet) and microtubules (mClover3-MAP4-MTBD), combined with our established 3D isosurface reconstruction and volume-tracking approaches to quantify chromosome dynamics (Dunkley and Mogessie, 2023; Leem et al., 2025). CENP-A TRIM-Away oocytes (using the commercially available CENP-A antibody, Ab2) failed to properly congress chromosomes, resulting in persistent scattering and frequent misalignment at metaphase (Figs. 3, f and g; and Videos 3 and 4). In control oocytes, chromosomes transiently disperse along the spindle axis following germinal vesicle breakdown (GVBD) before efficiently congressing at the metaphase plate (Mogessie et al., 2018). Consistent with this, 3D tracking of chromosome volumes showed that chromosomes aligned within ∼1.5 h after GVBD in controls (Fig. 3, f and g). In contrast, CENP-A TRIM-Away severely impaired congression, with chromosomes remaining dispersed even 2.5 h after GVBD, when controls had completed alignment. Using established quantitative criteria to assess alignment (Mogessie, 2020; Mogessie and Schuh, 2017), 85% of CENP-A TRIM-Away oocytes displayed misaligned chromosomes (Fig. 3 h), whereas none were observed in controls. Consistent with this, 19/23 CENP-A–depleted oocytes contained at least one misaligned chromosome and 14/23 contained two or more (Fig. 3 i), compared with 0/20 in controls. Chromosome segregation was also defective: although control oocytes completed chromosome separation within 12 min of anaphase onset, 16/23 CENP-A–depleted oocytes exhibited lagging chromosomes (Fig. 3, j and k). Notably, despite these frequent chromosome congression and segregation defects, CENP-A–depleted oocytes progressed through meiosis I with similar efficiency to controls (Fig. S3 d). Because CENP-A is required for kinetochore assembly (Fachinetti et al., 2013; McKinley and Cheeseman, 2016; Mellone and Fachinetti, 2021; Monda and Cheeseman, 2018), which coordinates SAC signaling (Musacchio, 2015; Musacchio and Salmon, 2007), its depletion is expected to compromise checkpoint function. Consistent with this, CENP-A TRIM-Away exhibited a shorter interval between GVBD and anaphase onset through meiosis I (Fig. S3 e). We recapitulated these chromosomal phenotypes using our custom-made CENP-A antibody Ab1 for TRIM-Away–mediated degradation (Fig. S3, f–m; and Videos 5 and 6). Together, these results show that CENP-A is required for accurate chromosome congression, alignment, and segregation during meiosis I in mouse oocytes, supporting a functional requirement for CENP-A that differs from its role in mitosis (Hoffmann et al., 2016).

**Video 3.** **CENP-A TRIM-Away using CENP-A Ab2–Control.** Time-lapse movie of the meiotic division from GVBD to metaphase II in a control TRIM-Away oocyte. Microtubules (gray) are labeled with mClover3-MAP4, and chromosomes (magenta) are labeled with H2B-mScarlet.

**Video 4.** **CENP-A TRIM-Away using CENP-A Ab2–CENP-A Ab2.** Time-lapse movie of the meiotic division from GVBD to metaphase II in a CENP-A TRIM-Away oocyte (commercial antibody). Microtubules (gray) are labeled with mClover3-MAP4, and chromosomes (magenta) are labeled with H2B-mScarlet.

**Video 5.** **CENP-A TRIM-Away using CENP-A Ab1–Control.** Time-lapse movie of the meiotic division from GVBD to metaphase II in a control TRIM-Away oocyte. Microtubules (gray) are labeled with mClover3-MAP4, and chromosomes (magenta) are labeled with H2B-mScarlet.

**Video 6.** **CENP-A TRIM-Away using CENP-A Ab1–CENP-A.** Time-lapse movie of the meiotic division from GVBD to metaphase II in a CENP-A Ab1 TRIM-Away oocyte. Microtubules (gray) are labeled with mClover3-MAP4, and chromosomes (magenta) are labeled with H2B-mScarlet.

**Figure S4.**
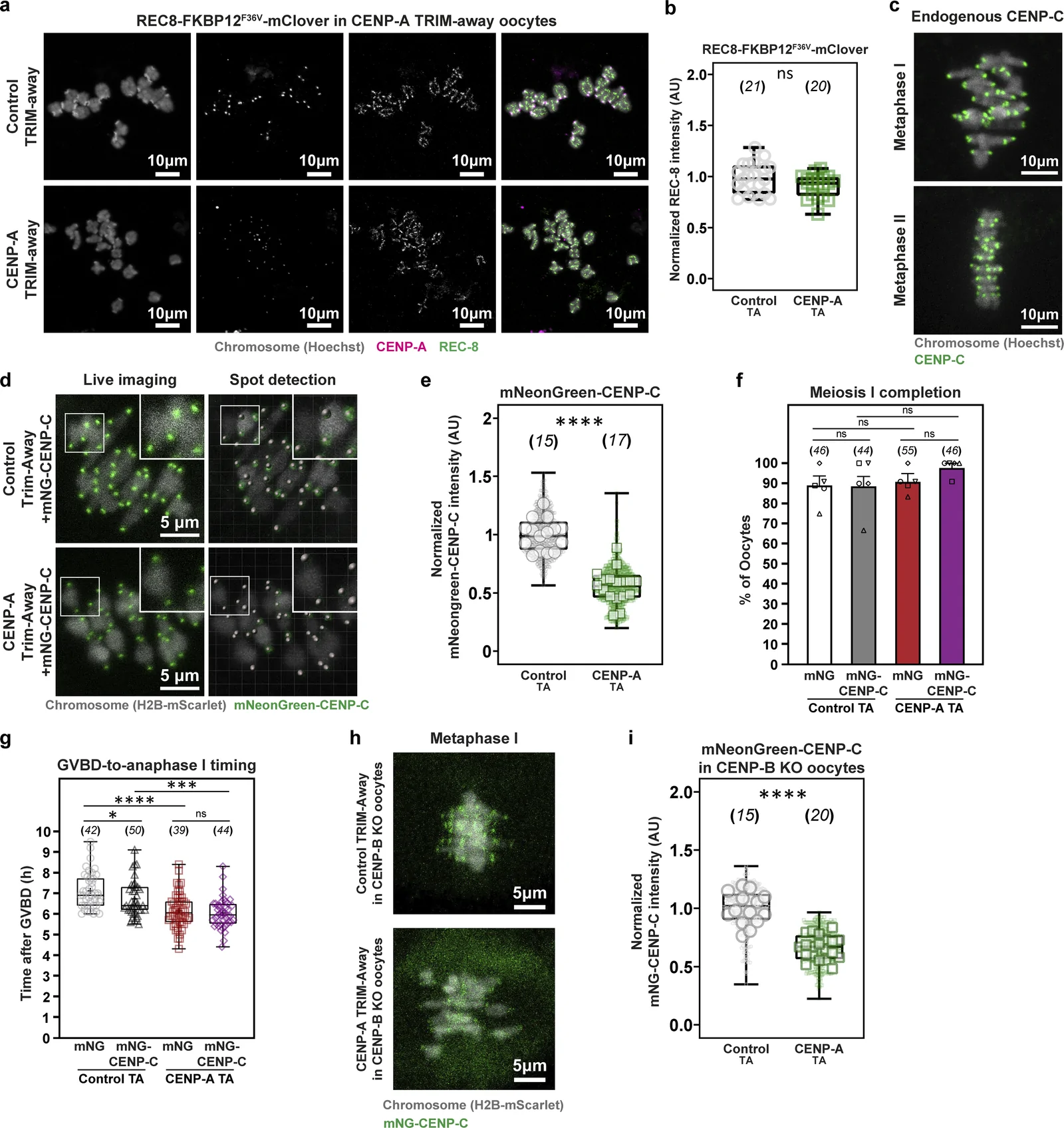
**Effects of CENP-A depletion on centromeric REC8 and CENP-C recruitment and meiotic progression in control and CENP-B KO oocytes. (a)** Representative maximum-intensity projections of immunofluorescence images showing chromosomes (Hoechst, gray), CENP-A (magenta), and REC8 (REC8-FKBP12^F36V^-mClover, green) in metaphase I chromosome spreads from control and CENP-A TRIM-Away oocytes. **(b)** Quantification of normalized REC8-FKBP12^F36V^-mClover fluorescence intensity in control and CENP-A TRIM-Away oocytes. Each circle represents one oocyte. Data are from three independent experiments. **(c)** Representative maximum-intensity projections of immunofluorescence images showing endogenous CENP-C (green) and chromosomes (Hoechst, gray) in intact metaphase I oocytes and metaphase II eggs. **(d)** Representative live imaging of chromosomes and kinetochores (H2B-mScarlet and mNG-CENP-C, respectively, left) and corresponding spot detection used for quantification (right) in control and CENP-A TRIM-Away oocytes expressing mNG-CENP-C during prometaphase I. Insets show magnified regions. **(e)** Quantification of mNG-CENP-C fluorescence integrated density. Each small circle and square represents an individual centromere, and larger circles and squares represent the mean per oocyte. Data are from three independent experiments. ****P < 0.0001. **(f)** Proportion of oocytes completing meiosis I. Each circle represents the mean of an independent experiment. Data are from three independent experiments. **(g)** Time from GVBD to anaphase I onset for individual oocytes. Data are from three independent experiments.*P = 0.0337, ***P = 0.0002, ****P < 0.0001. **(h)** Representative maximum-intensity projections of live imaging showing chromosomes and kinetochores in control and CENP-A (Ab2) TRIM-Away CENP-B KO oocytes expressing mNG-CENP-C during prometaphase I. **(i)** Quantification of mNG-CENP-C fluorescence intensity in control and CENP-A TRIM-Away CENP-B KO oocytes. Each small circle and square represents an individual centromere, and larger circles and squares represent the mean per oocyte. The number of oocytes analyzed per condition is indicated in brackets. Data are from three independent experiments. ****P < 0.0001. Statistical significance was evaluated using two-tailed Welch’s *t* test (b, e, and g [Control TRIM-Away+mNG vs Control TRIM-Away+mNG-CENP-C], i), two-tailed Mann–Whitney test (g [Control TRIM-Away+mNG vs CENP-A TRIM-Away+mNG, Control TRIM-Away+mNG-CENP-C vs CENP-A TRIM-Away+mNG-CENP-C, CENP-A TRIM-Away+mNG vs CENP-A TRIM-Away+mNG-CENP-C]), or Fisher’s exact test (f). The number of oocytes analyzed per condition is indicated in brackets. mNG, mNeonGreen.

To determine whether chromosome segregation defects after CENP-A degradation are due to cohesion loss, we measured cohesin levels in control and CENP-A TRIM-Away oocytes using REC8-FKBP12^F36V^-mClover3 mice, where REC8 cohesin can be detected with a GFP antibody (Leem et al., 2025). Immunofluorescence analysis showed similar REC8 localization along chromosome arms and centromeres in both control and CENP-A TRIM-Away oocytes (Fig. S4 a). Quantification of REC8 signal intensity showed no significant difference between the conditions (Fig. S4 b), indicating that acute CENP-A depletion does not measurably alter cohesin levels. These results suggest that the observed chromosome segregation defects are unlikely to result from cohesion loss and instead point to a cohesion-independent role for centromere weakening in meiotic errors.

### CENP-A disruption reduces CENP-C stability and impairs chromosome segregation in mammalian oocytes

**Figure 4.**
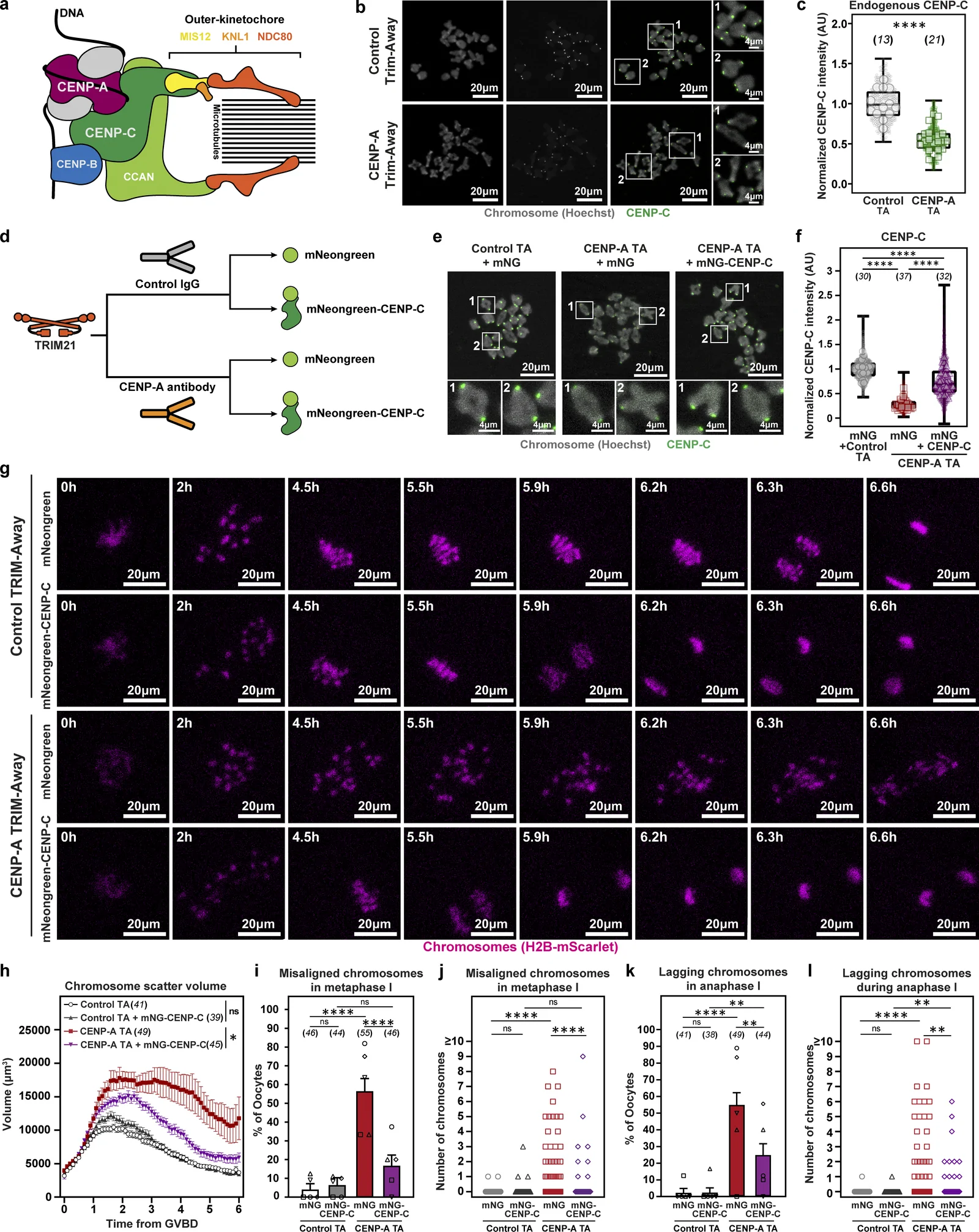
**Exogenous CENP-C expression rescues chromosome segregation defects induced by CENP-A depletion. (a)** Schematic illustration of the kinetochore–microtubule interface highlighting CENP-C as the link between the CENP-A chromatin and the outer kinetochore that engages microtubules. CCAN, constitutive centromere-associated network. Not drawn to scale. **(b)** Representative maximum-intensity projection immunofluorescence images of centromeres (CENP-C) and chromosomes (Hoechst) in chromosome spreads from control IgG (control) and CENP-A (Ab2) TRIM-Away oocytes. Boxed regions indicate areas shown at higher magnification. **(c)** Quantification of normalized integrated fluorescence density of endogenous centromeric CENP-C signals in b. Each small circle or square represents a single centromere, and each large circle or square represents the mean of a single oocyte. Box plots show median, interquartile range, and minimum to maximum values. Data are from three independent experiments. ****P < 0.0001. **(d)** Schematic of rescue strategy. Prophase-arrested oocytes were injected with TRIM21 mRNA and either control IgG or CENP-A (Ab2) antibody, together with mNG or mNG-CENP-C, then released from prophase arrest for live imaging. **(e)** Representative maximum-intensity projection immunofluorescence images of CENP-C and chromosomes (Hoechst) in metaphase I chromosome spreads from control TRIM-Away oocytes expressing mNG and CENP-A (Ab2) TRIM-Away oocytes expressing either mNG or mNG-CENP-C. Boxed regions indicate areas shown at higher magnification. **(f)** Quantification of the normalized integrated fluorescence density of centromeric CENP-C signals in e. Each small circle, square, or triangle represents a single centromere, and each large circle, square, or triangle represents the mean of a single oocyte. Box plots show the median, interquartile range, and the minimum and maximum values. Data are from four independent experiments. ****P < 0.0001. **(g)** Representative stills from 6-min time-lapse movies starting at GVBD showing chromosomes (H2B-mScarlet) in control and CENP-A (Ab2) TRIM-Away oocytes expressing either mNG or mNG-CENP-C during meiosis I. **(h)** Chromosome scattering volumes quantified from g over 6 h from GVBD. Lines indicate mean values, and error bars represent the SEM. Data are from five independent experiments. Total oocyte numbers are indicated in brackets. *P = 0.0167. **(i and j)** Proportion of oocytes with misaligned chromosomes (i) and number of misaligned chromosomes per oocyte (j), identified in (g), at metaphase I (final frame before anaphase onset). In (i), error bars represent the SEM and black circles indicate the mean of each independent experiment. Data are from five independent experiments. ****P < 0.0001 (**k and l)** Proportion of oocytes with severely lagging chromosomes (k) and number of severely lagging chromosomes per oocyte (l), identified in g, during anaphase I. Severely lagging chromosomes were defined as those persisting 18 min after anaphase onset, corresponding to three imaging frames. In (k), error bars represent the SEM and black circles indicate the mean of each independent experiment. Data are from five independent experiments. In k, control TRIM-Away + mNG-CENP-C versus CENP-A TRIM-Away + mNG-CENP-C, **P = 0.0044; CENP-A TRIM-Away + mNG versus CENP-A TRIM-Away + mNG-CENP-C, **P = 0.0057; other indicated comparisons, ****P < 0.0001. In l, control TRIM-Away + mNG-CENP-C versus CENP-A TRIM-Away + mNG-CENP-C, **P = 0.0024; CENP-A TRIM-Away + mNG versus CENP-A TRIM-Away + mNG-CENP-C, **P = 0.0013; other indicated comparisons, ****P < 0.0001. Statistical significance was evaluated using two-tailed Welch’s *t* tests (c and f, control TRIM-Away + mNG versus CENP-A TRIM-Away + mNG-CENP-C), two-tailed Mann–Whitney tests (f, control TRIM-Away + mNG versus CENP-A TRIM-Away + mNG and CENP-A TRIM-Away + mNG versus CENP-A TRIM-Away + mNG-CENP-C; j and l), mixed-effects analysis (h), or Fisher’s exact tests (i and k). The number of oocytes analyzed per condition is indicated in brackets. mNG, mNeonGreen.

The chromosome congression and segregation defects seen in CENP-A–depleted oocytes indicate compromised kinetochore function. CENP-A serves as a recruitment platform for the inner kinetochore protein CENP-C (Fachinetti et al., 2013; McKinley and Cheeseman, 2016; Mellone and Fachinetti, 2021), which seeds assembly of the CCAN and supports the formation of outer kinetochore structures essential for microtubule attachment (Klare et al., 2015; McKinley and Cheeseman, 2016; Monda and Cheeseman, 2018) (Fig. 4 a). We therefore hypothesized that CENP-A loss weakens centromere identity, reduces CENP-C recruitment, and impairs kinetochore function during meiosis. Endogenous CENP-C was readily detected at homologous kinetochores in metaphase I and sister kinetochores in metaphase II (Fig. S4 c). Quantification of CENP-C in metaphase I chromosome spreads showed that TRIM-Away–mediated CENP-A degradation reduced kinetochore-associated CENP-C levels by ∼50% relative to controls (Fig. 4, b and c), consistent with observations in mitotic cells following CENP-A loss (Fachinetti et al., 2013; Hoffmann et al., 2016).

To test whether reduced CENP-C underlies the meiotic defects observed in CENP-A–depleted oocytes, we next restored CENP-C levels and assessed chromosome dynamics (Fig. 4 d). Exogenous mNeonGreen-CENP-C expressed from microinjected mRNA localized to kinetochores in both control and CENP-A–depleted oocytes, although at reduced levels in CENP-A TRIM-Away oocytes (Fig. 4 e; and Fig. S4, d and e). Despite this, total kinetochore-associated CENP-C levels in CENP-A TRIM-Away oocytes increased from ∼25% to ∼65% of control levels following exogenous CENP-C expression (Fig. 4, e and f). This partial restoration improved chromosome behavior: excessive chromosome scattering was reduced, misalignment decreased to <20% of oocytes (6/46 with ≥2 misaligned chromosomes), and both the frequency and number of lagging chromosomes were reduced without affecting meiosis I completion (Fig. 4, g–l, Fig. S4 f; and Videos 7, 8, 9, and 10). However, meiotic timing remained accelerated, indicating incomplete rescue of kinetochore function, likely due to persistent defects in SAC signaling (Fig. S4 g). Together, these results support CENP-C recruitment as an important mechanism by which CENP-A maintains meiotic kinetochore function and suggest that additional CENP-A–independent pathways also contribute to CENP-C recruitment.

**Video 7.** **Exogenous expression of CENP-C in CENP-A TRIM-Away oocytes–Control + mNeonGreen.** Time-lapse movie of the meiotic division from GVBD to metaphase II in a control TRIM-Away oocyte expressing mNeonGreen. Chromosomes (magenta) are labeled with H2B-mScarlet.

**Video 8.** **Exogenous expression of CENP-C in CENP-A TRIM-Away oocytes–Control + mNeonGreen-CENP-C.** Time-lapse movie of the meiotic division from GVBD to metaphase II in a control TRIM-Away oocyte expressing mNeonGreen-CENP-C. Chromosomes (magenta) are labeled with H2B-mScarlet.

**Video 9.** **Exogenous expression of CENP-C in CENP-A TRIM-Away oocytes–CENP-A + mNeonGreen.** Time-lapse movie of the meiotic division from GVBD to metaphase II in a CENP-A TRIM-Away oocyte (commercial antibody) expressing mNeonGreen. Chromosomes (magenta) are labeled with H2B-mScarlet.

**Video 10.** **Exogenous expression of CENP-C in CENP-A TRIM-Away oocytes–CENP-A + mNeonGreen-CENP-C.** Time-lapse movie of the meiotic division from GVBD to metaphase II in a CENP-A TRIM-Away oocyte (commercial antibody) expressing mNeonGreen-CENP-C. Chromosomes (magenta) are labeled with H2B-mScarlet.

### CENP-B–mediated recruitment of CENP-C supports meiotic kinetochore function in the absence of CENP-A

**Figure 5.**
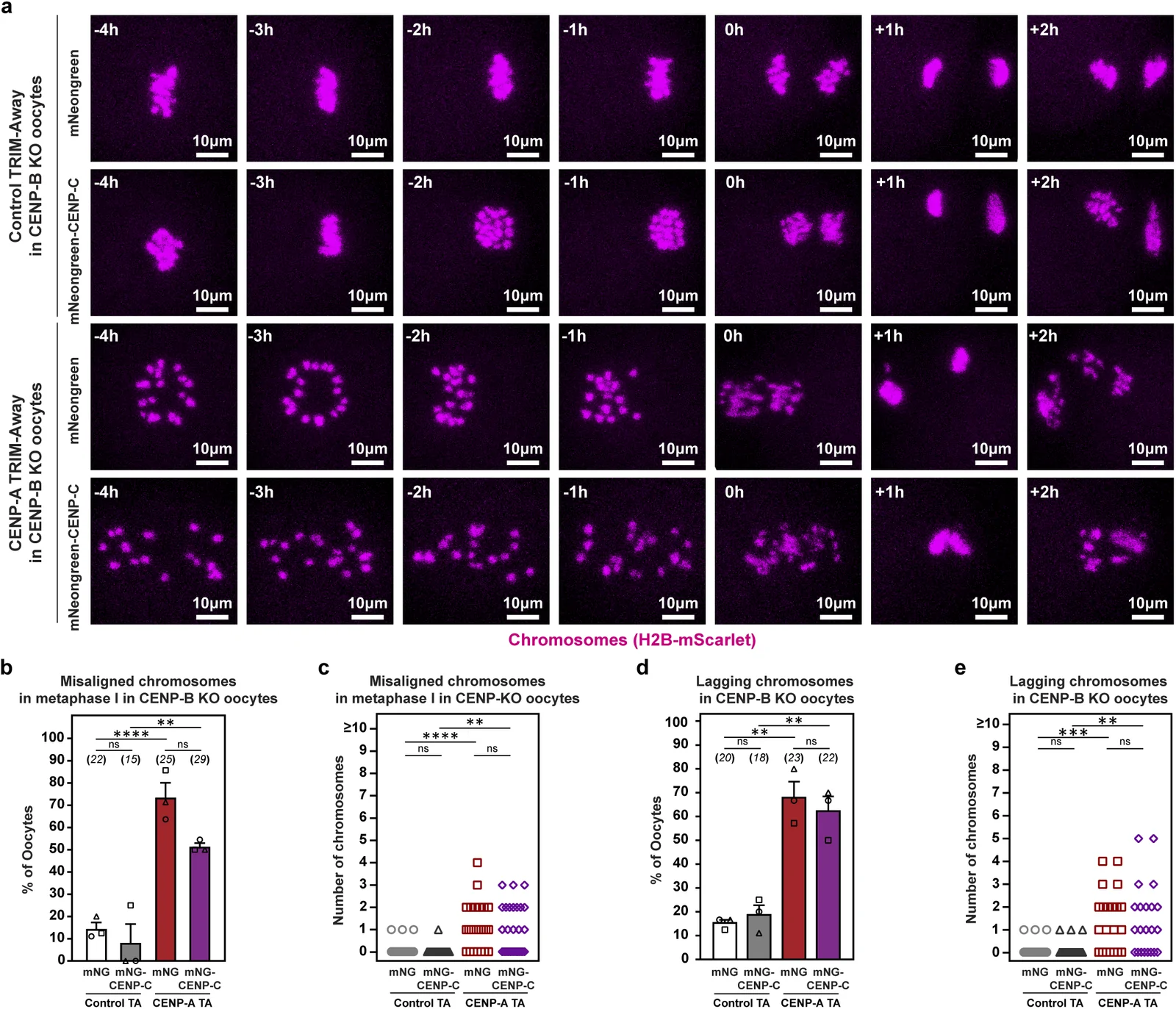
**CENP-B–mediated CENP-C recruitment supports meiotic kinetochore function in the absence of CENP-A. (a)** Representative stills from 6-min time-lapse movies showing chromosomes (H2B-mScarlet) in control and CENP-A (Ab2) TRIM-Away CENP-B KO oocytes expressing either mNG or mNG-CENP-C during meiosis I. Time 0 h corresponds to the onset of anaphase. **(b and c)** Proportion of oocytes with misaligned chromosomes (b) and number of misaligned chromosomes per oocyte (c), identified in a, at metaphase I (final frame before anaphase onset). In b, error bars represent the SEM, and black circles indicate the mean of each independent experiment. Data are from five independent experiments. (b) **P = 0.003, ****P < 0.0001; (c) ****P < 0.0001; **P = 0.003. **(d and e)** Proportion of oocytes with severely lagging chromosomes (d) and number of lagging chromosomes per oocyte (e), identified in a, during anaphase I. Lagging chromosomes are defined as chromosomes persisting 18 min after anaphase onset (three frames). In d, error bars represent the SEM and black circles indicate the mean of each independent experiment. Data are from five independent experiments. (d) Control TRIM-Away+mNG vs CENP-A TRIM-Away+mNG, **P = 0.0013, Control TRIM-Away+mNG-CENP-C vs CENP-A TRIM-Away+mNG-CENP-C, **P = 0.004; (e) Control TRIM-Away+mNG vs CENP-A TRIM-Away+mNG, ***P = 0.0002; Control TRIM-Away+mNG-CENP-C vs CENP-A TRIM-Away+mNG-CENP-C, **P = 0.001. Statistical significance was evaluated using two-tailed Welch’s *t* test (c [CENP-A TRIM-Away+mNG vs CENP-A TRIM-Away+mNG-CENP-C]), two-tailed Mann–Whitney test (c [Control TRIM-Away+mNG vs Control TRIM-Away+mNG-CENP-C, Control TRIM-Away+mNG vs CENP-A TRIM-Away+mNG, Control TRIM-Away+mNG-CENP-C vs CENP-A TRIM-Away+mNG-CENP-C], e), or Fisher’s exact test (b and d). The number of oocytes analyzed per condition is indicated in brackets. mNG, mNeonGreen.

Our data indicate that CENP-C can be stabilized at oocyte centromeres in a CENP-A–independent manner during meiosis I. In mitotic cells, faithful chromosome segregation in the absence of CENP-A depends on CENP-B bound to CENP-B boxes within the centromeric DNA, which preserves a functional pool of CENP-C through direct interaction (Fachinetti et al., 2013; Fachinetti et al., 2015; Fujita et al., 2015; Hoffmann et al., 2016). To test whether CENP-B is required for CENP-C–mediated rescue of chromosome alignment and segregation defects in the absence of CENP-A, we performed TRIM-Away–mediated degradation of CENP-A in oocytes isolated from CENP-B knockout (KO) mice (Kumon et al., 2021). In contrast to CENP-A TRIM-Away oocytes in a wild-type background, where exogenous CENP-C localized to kinetochores (Fig. 4 e and Fig. S4 d), exogenously expressed mNeonGreen-CENP-C failed to efficiently localize to centromeres in CENP-B KO oocytes following CENP-A TRIM-Away (Fig. S4, h and i). Consistent with this, exogenous mNeonGreen-CENP-C did not rescue chromosome alignment or segregation defects in the absence of CENP-B. Here, we observed that >50% of CENP-A–depleted oocytes from CENP-B KO mice exhibited at least one misaligned chromosome, and >60% displayed lagging chromosomes during anaphase I, regardless of exogenous mNeonGreen-CENP-C expression (Fig. 5, a–e; and Videos 11, 12, 13, and 14). Importantly, as previously observed (Fig. 4, g–l), the overexpression of mNeonGreen-CENP-C alone did not induce chromosome alignment or segregation defects (Fig. 5, a–e), suggesting that the observed phenotypes are specific to loss of CENP-A and CENP-B rather than exogenous CENP-C expression. Together, these results support a genetic epistasis model in which CENP-B promotes the CENP-A–independent recruitment or retention of CENP-C at centromeres, thereby sustaining kinetochore function. Although demonstrated here with exogenous CENP-C, this mechanism is also likely to support the residual endogenous CENP-C pool after CENP-A depletion.

**Video 11.** **Exogenous expression of CENP-C in CENP-B KO oocytes–Control + mNeonGreen.** Time-lapse movie of the meiotic division from GVBD to metaphase II in a control TRIM-Away oocyte expressing mNeonGreen. Chromosomes (magenta) are labeled with H2B-mScarlet.

**Video 12.** **Exogenous expression of CENP-C in CENP-B KO oocytes–Control + mNeonGreen-CENP-C.** Time-lapse movie of the meiotic division from GVBD to metaphase II in a control TRIM-Away oocyte expressing mNeonGreen-CENP-C. Chromosomes (magenta) are labeled with H2B-mScarlet.

**Video 13.** **Exogenous expression of CENP-C in CENP-B KO oocytes–CENP-A TRIM-Away + mNeonGreen.** Time-lapse movie of the meiotic division from GVBD to metaphase II in a CENP-A TRIM-Away oocyte expressing mNeonGreen. Chromosomes (magenta) are labeled with H2B-mScarlet.

**Video 14.** **Exogenous expression of CENP-C in CENP-B KO oocytes–CENP-A TRIM-Away + mNeonGreen-CENP-C.** Time-lapse movie of the meiotic division from GVBD to metaphase II in a CENP-A TRIM-Away oocyte expressing mNeonGreen-CENP-C. Chromosomes (magenta) are labeled with H2B-mScarlet.

### Increasing CENP-C levels partially improves chromosome alignment in oocytes from moderately aged mice

The convergence of centromere weakening and cytoskeletal disorganization with age suggests that multiple cohesion-independent defects underlie oocyte aneuploidy. To determine whether centromere weakening underlies meiotic errors, we tested whether restoring kinetochore function could improve chromosome dynamics in aged oocytes. Building on our finding that increasing CENP-C level rescues chromosome alignment and segregation in CENP-A–depleted oocytes (Fig. 4), we examined whether exogenous CENP-C expression could similarly enhance meiotic fidelity in naturally aged oocytes, which exhibit reduced levels of both CENP-A and CENP-C (Smoak et al., 2016; Yin et al., 2023) (Fig. 2, a and b).

**Figure S5.**
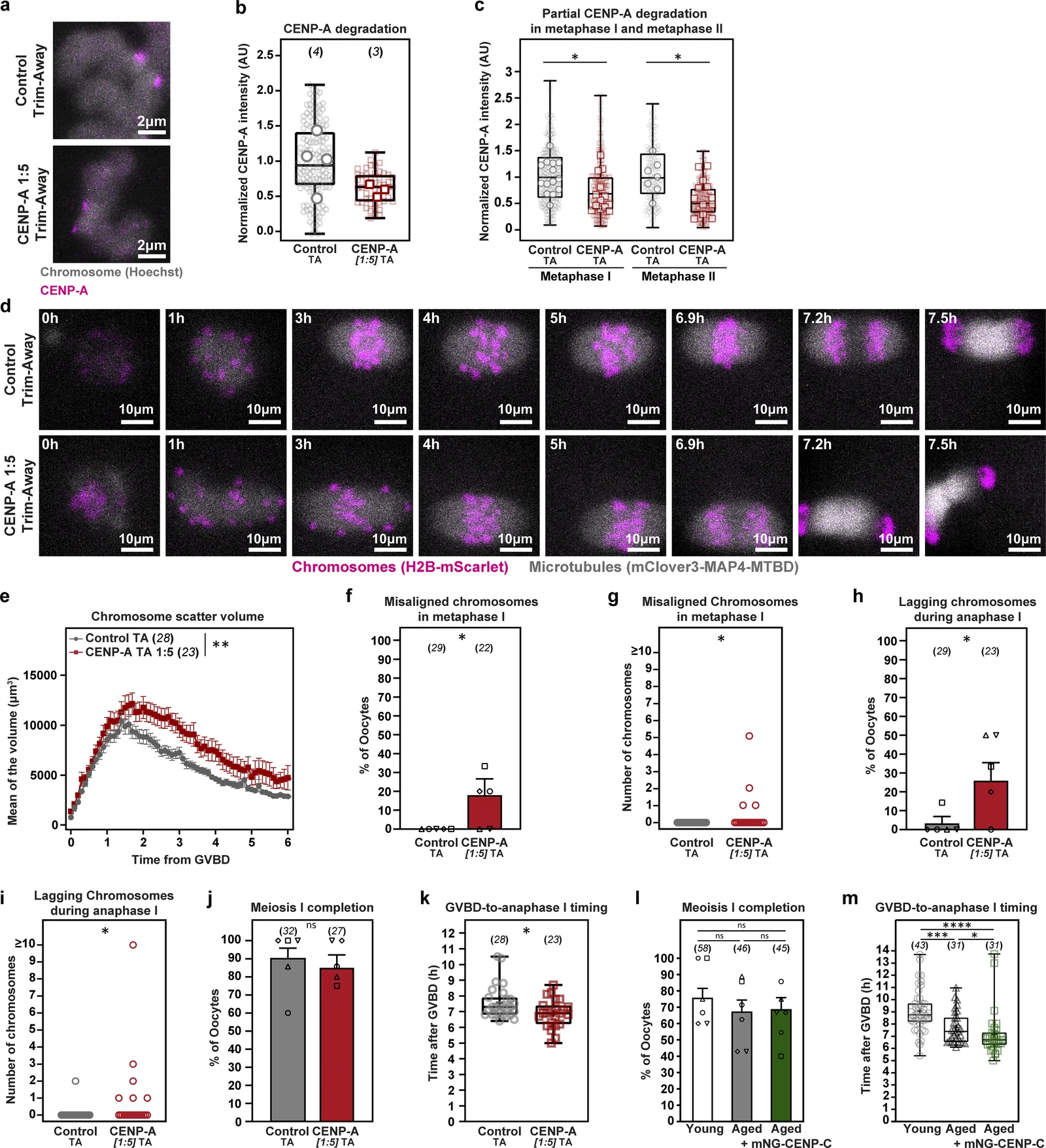
**Partial CENP-A depletion induces mild chromosomal abnormalities during meiosis I. (a)** Representative maximum-intensity projections of immunofluorescence images showing chromosomes (Hoechst, gray) and CENP-A (magenta) in metaphase II chromosome spreads from control and partial CENP-A TRIM-Away oocytes (1:5 dilution of Ab2). **(b)** Quantification of normalized CENP-A fluorescence integrated density in metaphase II chromosome spreads from control and partial CENP-A TRIM-Away eggs. Each small circle and square represents an individual centromere, and larger circles and squares represent the mean per egg. Data are from one experiment. **(c)** Quantification of normalized CENP-A fluorescence integrated density in metaphase I and metaphase II chromosome spreads from control and CENP-A TRIM-Away oocytes and eggs. Each symbol represents a centromere. Data are from three independent experiments. Metaphase I, *P = 0.0186; Metaphase II, *P = 0.0467. **(d)** Representative stills from time-lapse imaging of chromosomes and microtubules in control and partial CENP-A TRIM-Away oocytes during meiosis I. Time is indicated relative to GVBD. **(e)** Mean chromosome scatter volume over time following GVBD in control and partial CENP-A TRIM-Away oocytes. Error bars represent the SEM. Data are from five independent experiments. **P = 0.0063. **(f and g)** Frequency of oocytes with misaligned chromosomes (f) and number of misaligned chromosomes per oocyte (g) at metaphase I, defined as the final frame prior to anaphase onset. Data are from five independent experiments. (f) *P = 0.0293, (g) *P = 0.0293. **(h and i)** Frequency of oocytes with lagging chromosomes (h) and number of lagging chromosomes per oocyte (i) during anaphase I. Lagging chromosomes were defined as those trailing behind the segregating chromosome mass 18 min (three frames) after anaphase onset. Data are from five independent experiments. **(h)** *P = 0.0158, (i) *P = 0.0291. **(j)** Proportion of oocytes completing meiosis I. Each circle represents the mean of an independent experiment. Data are from five independent experiments. **(k)** Time from GVBD to anaphase I onset for individual oocytes. Data are from five independent experiments. *P = 0.0216. **(l)** Proportion of oocytes completing meiosis I. Each circle represents the mean of an independent experiment. Data are from six independent experiments. **(m)** Time from GVBD to anaphase I onset for individual oocytes. Data are from six independent experiments. *P = 0.0106, ***P = 0.0004, ****P < 0.0001. In f, h, j, and l, symbols represent the means of independent experiments and error bars indicate the SEM. Statistical significance was evaluated using two-tailed Welch’s *t* tests (c and m [Young+mNG vs Aged + mNG]), two-tailed Mann–Whitney tests (g, i, k, and m [Young+mNG vs Aged+mNG-CENP-C, Aged+mNG vs Aged+mNG-CENP-C]), or Fisher’s exact tests (f, h, j, and l). The number of oocytes analyzed per condition is indicated in brackets. mNG, mNeonGreen.

We first tested whether partial CENP-A degradation, mimicking the reduction observed in oocytes from reproductively older females, perturbs meiosis I progression in young oocytes. To induce partial CENP-A degradation, we performed TRIM-Away with a fivefold lower concentration of the Ab2 antibody. Under these conditions, CENP-A levels at kinetochores were reduced by ∼50% relative to control oocytes (Fig. S5, a and b), compared with the ∼80% reduction achieved using the higher concentrations of Ab1 and Ab2 antibodies (Fig. 3, b and e). This reduction was already present in metaphase I oocytes as, under the same conditions, CENP-A levels at the kinetochore of homologous chromosomes were also reduced by a similar amount—around 50%—relative to control metaphase I oocytes (Fig. S5 c). Chromosome scattering analyses showed that partial CENP-A depletion caused milder chromosome congression defects (Fig. S5, d and e; and Videos 15 and 16). These phenotypes were accompanied by chromosome alignment and segregation errors (Fig. S5, f–i). Consistent with impaired kinetochore assembly and likely defective SAC signaling, partial CENP-A depletion did not delay the completion of meiosis I (Fig. S5 j) and slightly accelerated the time from GVBD to anaphase onset (Fig. S5 k).

**Video 15.** **Partial CENP-A TRIM-Away using CENP-A Ab2–Control.** Time-lapse movie of the meiotic division from GVBD to metaphase II in a control TRIM-Away oocyte. Microtubules (gray) are labeled with mClover3-MAP4, and chromosomes (magenta) are labeled with H2B-mScarlet.

**Video 16.** **Partial CENP-A TRIM-Away using CENP-A Ab2–CENP-A Ab2.** Time-lapse movie of the meiotic division from GVBD to metaphase II in a 1:5-dilution CENP-A Ab2 TRIM-Away oocyte. Microtubules (gray) are labeled with mClover3-MAP4, and chromosomes (magenta) are labeled with H2B-mScarlet.

**Figure 6.**
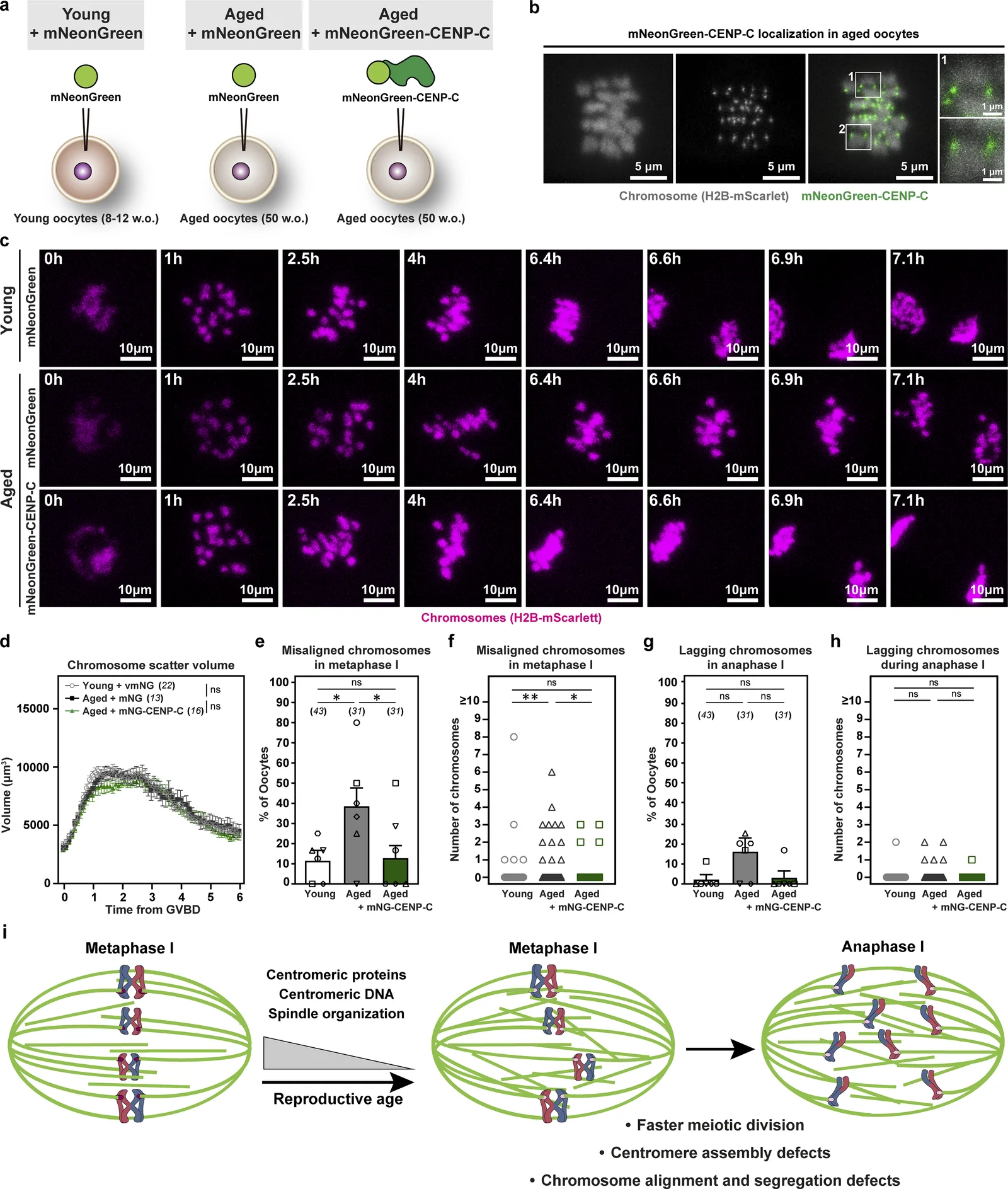
**CENP-C partially improves chromosome alignment in oocytes from moderately aged mice. (a)** Schematic of experimental design for exogenous expression of mNG-CENP-C in aged oocytes. Young prophase-arrested oocytes were injected with mNG mRNA alone as “young” control. Aged prophase-arrested oocytes were injected with either mNG or mNG-CENP-C mRNA, released from prophase arrest, and imaged live. **(b)** Representative maximum-intensity projection live image of chromosomes (H2B-mScarlet, gray) and kinetochore (mNG-CENP-C, green) in aged metaphase I oocytes expressing mNG-CENP-C. Boxed regions indicate areas shown at higher magnification. **(c)** Representative stills from time-lapse movies starting at GVBD showing chromosomes (H2B-mScarlet) in young oocytes expressing mNG and aged oocytes expressing either mNG or mNG-CENP-C mRNA during meiosis I. **(d)** Chromosome scattering volumes quantified from c over 6 h from GVBD. Lines indicate mean values, and error bars represent the SEM. Data are from six independent experiments. **(e and f)** Proportion of oocytes with misaligned chromosomes (e) and number of misaligned chromosomes per oocyte (f), identified in c, at metaphase I (final frame before anaphase onset). In e, error bars represent the SEM and circles indicate the mean of each independent experiment. Data are from six independent experiments. (e) Young+mNG vs Aged+mNG, *P = 0.0106, Aged+mNG vs Aged+mNG-CENP-C, *P = 0.0402. (f) **P = 0.0058, *P = 0.0268. **(g and h)** Proportion of oocytes with severely lagging chromosomes (g) and number of severely lagging chromosomes per oocyte (h), identified in c, during anaphase I. Severely lagging chromosomes are defined as those persisting for at least 18 min after anaphase onset (three frames). In g, error bars represent the SEM and circles indicate the mean of each independent experiment. Data are from six independent experiments. **(i)** Model illustrating how advancing maternal age reduces centromeric protein levels and alters spindle organization, leading to chromosome alignment and segregation defects in mammalian oocytes. In e and g, each symbol represents the mean of one independent experiment, comprising oocytes collected from one young mouse or at least three aged mice. In f and h, each symbol represents an individual oocyte. The number of oocytes analyzed in each independent experiment is provided in the source data. Statistical significance was evaluated using the two-tailed Mann–Whitney test (f and h), mixed-effects analysis (d), or Fisher’s exact test (e and g). The number of oocytes analyzed per condition is indicated in brackets. mNG, mNeonGreen.

Based on these findings, we analyzed cells that had not yet developed severe chromosome alignment or segregation defects, allowing a more rigorous evaluation of whether exogenous CENP-C expression could improve meiotic fidelity. Live imaging showed that mNeonGreen-CENP-C expressed from microinjected mRNA (Fig. 6 a) localized to kinetochores in aged oocytes, confirming efficient recruitment of the exogenous protein to centromeric chromatin (Fig. 6 b). High-resolution live imaging combined with 3D chromosome tracking showed that aged oocytes progressively congressed initially scattered chromosomes toward the spindle equator during meiosis I but frequently failed to achieve complete alignment (Fig. 6, c–e; and Videos 17 and 18). Whereas 12% of young oocytes displayed chromosome alignment defects, this frequency increased to 39% in oocytes from moderately aged mice (Fig. 6, c and e). The expression of mNeonGreen-CENP-C reduced the incidence of chromosome misalignment in aged oocytes to 13% and also decreased the number of misaligned chromosomes per oocyte (Fig. 6, c, e, and f; and Videos 17, 18, and 19). Since these moderately aged oocytes had not yet exhibited a robust increase in severely lagging chromosomes during anaphase I, exogenous CENP-C did not produce a detectable change in this outcome (Fig. 6, c, g, and h). Aged oocytes, with or without exogenous CENP-C expression, completed meiosis I at rates similar to young oocytes (Fig. S5 l). Despite their elevated frequency of chromosome misalignment, aged oocytes progressed through meiosis I more rapidly than young oocytes. Exogenous CENP-C expression did not alter this accelerated timing phenotype (Fig. S5 m). Thus, under these experimental conditions, exogenous CENP-C improves the emerging chromosome alignment defect associated with reproductive aging but does not measurably alter chromosome segregation outcomes.

**Video 17.** Time-lapse movie of the meiotic division from GVBD to metaphase II in a young oocyte expressing mNeonGreen. Chromosomes (magenta) are labeled with H2B-mScarlet.

**Video 18.** **Exogenous expression of CENP-C in aged oocytes–Aged + mNeonGreen.** Time-lapse movie of the meiotic division from GVBD to metaphase II in an aged oocyte expressing mNeonGreen. Chromosomes (magenta) are labeled with H2B-mScarlet.

**Video 19.** **Exogenous expression of CENP-C in aged oocytes–Aged + mNeonGreen-CENP-C.** Time-lapse movie of the meiotic division from GVBD to metaphase II in an aged oocyte expressing mNeonGreen-CENP-C. Chromosomes (magenta) are labeled with H2B-mScarlet.

Together with our previous evidence implicating CENP-A loss and spindle actin disruption in age-related oocyte aneuploidy (Dunkley and Mogessie, 2023; Leem et al., 2025), these results support a model in which centromere weakening acts in parallel with cohesion loss and cytoskeletal dysfunction to compromise meiotic fidelity during reproductive aging.

## Discussion

Maternal age–related cohesion depletion is recognized as a major cause of aneuploidy in oocytes (Herbert et al., 2015). However, the sudden rise in oocyte aneuploidy at advanced reproductive ages suggests that additional mechanisms are at play. Our findings support a previously unrecognized role for centromere weakening in meiotic errors, consistent with emerging evidence that centromere composition changes with age (Smoak et al., 2016; Yin et al., 2023; Zielinska et al., 2019). Direct evidence for this comes from an engineered oocyte aging system in which partial CENP-A depletion synergizes with cohesion loss to accelerate premature chromosome separation, supporting a model in which centromere weakening acts as a cohesion-independent driver of aneuploidy (Leem et al., 2025) (Fig. 6 i). Our complementation experiments show that the improvement of the CENP-A depletion phenotype by exogenous CENP-C requires CENP-B, supporting a role for CENP-B-box–containing centromeric DNA in maintaining CENP-C recruitment. In humans, CENP-B box abundance and distribution vary across chromosomes (Altemose et al., 2022; Dumont et al., 2020; Earnshaw et al., 1989), and centromeric and pericentromeric sequences show substantial divergence (Packiaraj and Thakur, 2024). Although CENP-A declines uniformly with age, this sequence heterogeneity raises the possibility that subtle variation in centromeric DNA influences CENP-A occupancy and contributes to chromosome-specific segregation errors (Dumont et al., 2020).

A recent work showed that paternal centromeres, which enter the zygote with substantially lower CENP-A levels than maternal centromeres, preferentially recruit maternal CENP-C to promote CENP-A restoration before the first embryonic mitosis (Tower et al., 2026). These findings identify CENP-C as an instructive regulator of CENP-A replenishment and suggest that reduced CENP-C recruitment, rather than CENP-A loss alone, is a critical determinant of age-associated centromere dysfunction. Accordingly, exogenous CENP-C may improve chromosome alignment in aged oocytes not only by supporting kinetochore assembly downstream of the remaining CENP-A but also by promoting the restoration or stabilization of endogenous CENP-A. Because CENP-A incorporation was not measured after CENP-C expression in this study, this potential mechanism remains to be tested.

Our observation that both major and minor satellite FISH signals are reduced in aged oocytes suggests that even the most functionally conserved centromeric regions are vulnerable to maternal aging, positioning core centromere disruption as an underappreciated driver of age-related oocyte quality decline. The reduction of minor satellites, and consequently the CENP-B boxes needed to recruit CENP-B to centromeres, could also explain why CENP-C re-expression is not sufficient to fully restore chromosome alignment in aged oocytes compared with what is observed in CENP-A TRIM-Away oocytes. The decrease of both major and minor satellites raises an important mechanistic question about how these FISH signals become diminished. One possibility is that accumulated DNA damage in aged oocytes (Carroll and Marangos, 2013; Pelosi et al., 2015; Titus et al., 2013) leads to rearrangement or permanent loss of satellite sequences, consistent with evidence that centromeric DNA remains unstable even during prolonged oocyte quiescence (Saayman et al., 2023; Scelfo and Fachinetti, 2023). Alternatively, a reduced signal could reflect altered chromatin accessibility or compaction that masks these sequences without changing the underlying DNA. Distinguishing between these scenarios will be important for determining whether persistent DNA damage drives satellite signal loss and enables transmission of centromeric defects across generations, or whether epigenetic alterations underlie this loss and permit resetting during early embryonic reprogramming.

In parallel with centromere weakening, our ultrastructural analyses suggest that microtubule orientation, interaction, and geometry are altered in aged oocytes. These structural differences could affect spindle function through mechanisms beyond end-on kinetochore attachment and may reflect disruption of the spindle as a mechanically integrated network. One possibility is that misoriented microtubules generate off-axis forces that contribute to chromosome misalignment even in the presence of kinetochore–microtubule attachments. Disrupted microtubule interactions may weaken tension-dependent error correction, which relies on coordinated spindle architecture to destabilize incorrect attachments (Musacchio, 2015; Musacchio and Salmon, 2007; Vallot et al., 2018). Reduced interaction lengths and increased intermicrotubule angles may reduce mechanical coupling across the spindle, compromising coordinated chromosome movements (Shimamoto et al., 2011; Redemann et al., 2017; Vukušić et al., 2017; Vukušić et al., 2019; Kiewisz et al., 2022). Consistent with this view, ultrastructural analyses of spindles in *C. elegans* have shown that kinetochore–microtubules do not form continuous fibers extending from kinetochores to spindle poles, as depicted in classical models, but instead exist as shorter, cross-linked networks that collectively transmit forces across the spindle (Redemann et al., 2017). In this framework, misorientation and reduced bundling of chromosome-proximal microtubules, as observed here, are expected to disrupt force transmission and coordination across the spindle, even when end-on attachments are established. Although we did not reconstruct the entire meiotic spindle, our data are consistent with altered microtubule organization in aged oocytes that may reduce chromosome segregation fidelity.

These cytoskeletal defects may act alongside centromere weakening (Fig. 6 i). Chromosomes with reduced centromere strength are likely more susceptible to segregation errors in a spindle that generates abnormal forces. This, along with the observed reduction in minor satellites, may explain why CENP-C re-expression improves chromosome alignment in aged oocytes but does not fully restore meiotic fidelity, as the underlying spindle defects remain unresolved. In mitotic cells, similar spindle disorganization arises from defects in motor and nonmotor MAPs (Subramanian et al., 2010; Walczak and Heald, 2008). Although MAP function was not directly tested here, the structural parallels suggest that age-dependent changes in MAP expression, localization, or activity may contribute to spindle deterioration in oocytes. Consistently, recent proteomic analyses of oocyte aging identified changes in the abundance of several MAPs, including TACC3, CKAP5, and CLASP1 (Zhang et al., 2025). Together with our prior work showing that actin disruption exacerbates the effects of age-related cohesion loss (Dunkley and Mogessie, 2023), these observations identify cytoskeletal dysregulation as a candidate cohesion-independent contributor to reproductive decline.

In summary, our findings implicate age-related centromere weakening in meiotic errors in mammalian oocytes, acting in parallel with spindle disorganization to impair chromosome segregation. The uniform decline in centromeric proteins across all chromosomes suggests a genome-wide vulnerability to maternal aging, while cytoskeletal defects may introduce an independent layer of mechanical instability. This multifactorial model of oocyte aneuploidy (Fig. 6 i) suggests that effective future strategies to preserve oocyte quality during maternal aging would need to address centromere integrity, cytoskeletal architecture, and cohesion together, rather than targeting any single age-associated defects.

## Materials and methods

### Mouse oocyte isolation, maturation, culture, and microinjection

All protocols involving mice were approved by the Yale University Institutional Animal Care and Use Committee (approval number 2024-20408). Mice were housed under specific-pathogen-free conditions with a 12-h light–dark cycle. The mouse strains used in this study included 8- to 12-wk-old CD1 or C57BL/6 mice (young) and 8- to 9-mo-old CD1 or 10- to 11-mo-old C57BL/6 mice (aged). At least 2 CD1 mice were used per experiment. For the experiments shown in Fig. 6, one young C57BL/6 and at least 3 aged C57BL/6 mice were used per experiment.

Fully grown, prophase-arrested oocytes were collected from ovaries in M2 medium supplemented with dibutyryl cyclic AMP (dbcAMP) to maintain prophase arrest. Oocyte culture, *in vitro* maturation, and microinjection were performed according to our previously published open-access methods chapter (Mogessie, 2020), which provides extensive step-by-step protocols designed to facilitate implementation and reproducibility. Individual mRNAs were prepared at stock concentrations of 5–10 µg/μl, diluted 1:5 to 1:200, and microinjected at 4–6 pl per oocyte. Based on an estimated mouse oocyte volume of ∼200 pl (Jaffe et al., 2009), these microinjection parameters correspond to final intracellular mRNA concentrations of ∼0.5–60 ng/μl. Injected mRNAs were expressed for 3–4 h, while oocytes remained arrested at the prophase I germinal vesicle stage. Meiotic resumption was then induced by washing the oocytes into M2 medium without dbcAMP.

### REC8-FKBP12^F36V^-mClover3 and CENP-B KO mice

We previously generated and validated the REC8-FKBP12^F36V^-mClover3 knock-in line (Leem et al., 2025). Briefly, CRISPR-Cas9 was used to insert an FKBP12^F36V^-mClover3 cassette immediately upstream of the endogenous REC8 stop codon in C57BL/6J zygotes. Editing used the guide RNA 5′-GGT​TTA​ATG​AAC​TTG​CTC​AG-3′ and a 1.5-kb single-stranded DNA donor containing the knock-in cassette. We identified correctly targeted founders using PCR primers BM732 and BM733 for internal PCR, BM734 and BM729 for 5′ junction PCR, and BM730 and BM735 for 3′ junction PCR. We Sanger-sequenced the junction products to verify the insert and homology arms, and confirmed germline transmission by breeding. Homozygous animals were maintained as an independent colony and outcrossed to C57BL/6J every five generations. CENP-B KO mice were provided by the laboratory of Michael A. Lampson (University of Pennsylvania, Philadelphia, PA, USA). The Lampson laboratory originally generated and characterized this line (Kumon et al., 2021). Briefly, CRISPR-Cas9 genome editing targeting the DNA-binding domain of CENP-B introduced a 37-bp deletion that produces a premature stop codon and eliminates detectable CENP-B protein. Correct genotypes were identified by PCR using primers BM830 and BM831. Wild-type and mutant alleles were distinguished by the 37-bp difference in amplicon size.

### Generation of *in vitro* mRNA transcription constructs

All the oligonucleotide sequences used to generate the different constructs in this study are listed in Table S1. The pGEM-HE plasmid, originally generated and characterized in a previous study (Schuh and Ellenberg, 2007), was used as the backbone for *in vitro* transcription constructs in this study. pGEM-mScarlet-N for C-terminal fusion to mScarlet was generated by PCR linearization of pGEM-HE with primers BM489-BM490 and the Gibson assembly with the mScarlet ORF (GenBank: KY021423.1), amplified with primers BM491–BM492. pGEM-H2B-mScarlet was generated by inserting the KS109–KS110 amplified mouse H2B ORF (NM_001290380.1) into the EcoRI-XhoI site of pGEM-mScarlet-N. To produce pGEM-mClover3-MAP4-MTBD, the KS111–KS112 amplified mClover3 sequence (GenBank: KX987298.1) was inserted into the AflII-NcoI site of pGEM-mEGFP-MAP4-MTBD (Mogessie and Schuh, 2017), replacing mEGFP with mClover3. pGEM-SNAP-TRIM21, generated and validated in our previous study (Dunkley and Mogessie, 2023), encodes SNAP-tagged TRIM21 and was used as the template for producing capped SNAP-TRIM21 mRNA for TRIM-Away experiments. pGEM-mNeonGreen-N for C-terminal fusion to mNeonGreen was constructed via the Gibson assembly by inserting the BM766-BM767 amplified mNeonGreen sequence (GenBank: OK040229.1) into pGEM-HE, which was linearized with primers BM765-BM764. pGEM-mNeonGreen-CENP-C was created by inserting the mNeonGreen sequence amplified with TL14-TL15 into the linearized pGEM-CENP-C containing mouse CENP-C (XP_030109955.1) using the Gibson assembly. Capped mRNAs were synthesized using T7 polymerase (mMESSAGE mMACHINE Kit, Ambion). mRNA concentrations were measured with a NanoDrop spectrophotometer (Thermo Fisher Scientific).

### Fixation and CENP-A immunostaining of intact oocytes

Oocytes were fixed in a buffer containing 100 mM HEPES, 10 mM MgSO_4_, 50 mM EGTA, 0.5% (vol/vol) Triton X-100, and 2% (vol/vol) formaldehyde for 20–30 min at 37°C. Cells were then extracted in PBS supplemented with 0.25% (vol/vol) Triton X-100 for 10 min at room temperature, followed by overnight blocking in PBS with 3% (wt/vol) bovine serum albumin (BSA) at 4°C. Fixed oocytes were treated with λ-phosphatase (P0753S; New England Biolabs) for 2 h at 30°C, washed in PBS, and incubated with rabbit anti-CENP-A primary antibodies (rabbit monoclonal [1:200; C51A7; Cell Signaling] or purified custom rabbit polyclonal Ab1 [1:1,000; PAC-19283/19284; Pacific Immunology]) for 1.5 h at 37°C. After PBS washes, samples were incubated with Alexa Fluor 488–conjugated anti-rabbit secondary antibody (1:500, A11008; Invitrogen) for 1 h at 37°C. DNA was counterstained with Hoechst 33342 (5 μg/ml, 62249; Thermo Fisher Scientific) for 10 min at room temperature.

### Metaphase chromosome spreading, fixation, and immunostaining

The zona pellucida was removed from metaphase I oocytes or metaphase II–arrested eggs to enable efficient chromosome spreading by washing cells through three droplets of Tyrode’s solution (T1788; Sigma-Aldrich). Zona-free cells were then washed sequentially through several droplets of M2 medium and allowed to recover for at least 10 min at 37°C. For chromosome spreading, individual cells were pipetted onto wells of 15-well Multitest slides (096041505; MP Biomedicals) containing a water-based fixative solution composed of 1% (vol/vol) formaldehyde, 0.15% (vol/vol) Triton X-100, and 3% (vol/vol) DTT, adjusted to pH 9.2–9.4. Slides were incubated overnight at room temperature in an opaque humidified chamber. The following day, chromosome spreads were air-dried at room temperature and stored frozen until further processing.

Chromosome spreads were blocked in 3% (wt/vol) BSA (A9647; Sigma-Aldrich) in PBS for 30 min at room temperature in a humidified chamber. For CENP-A immunostaining, spreads were treated with λ-phosphatase (P0753S; New England Biolabs) at 30°C for 2 h, followed by PBS washes before antibody incubation. Spreads were then incubated with primary antibodies against CENP-A (rabbit monoclonal [1:200; C51A7; Cell Signaling] or purified custom rabbit polyclonal Ab1 [1:1,000; PAC-19283/19284; Pacific Immunology]) or rabbit polyclonal CENP-C antibody (Das et al., 2022) (1:10,000) for 1.5 h at 37°C. After three washes in PBS (10 min each), spreads were incubated with species-specific secondary antibodies diluted in blocking buffer (Alexa Fluor 488–conjugated anti-rabbit IgG, 1:500, A11008; Invitrogen; or Cy3-conjugated donkey anti-rabbit IgG [H+L], 1:500, Jackson ImmunoResearch) for 1 h at 37°C. DNA was counterstained with Hoechst 33342 (5 μg/ml, 62249; Thermo Fisher Scientific) for 30 min at room temperature.

Slides were washed in PBS and mounted in VECTASHIELD Antifade Mounting Medium (H-1000-10; 2BScientific), covered with 22 × 22 mm glass coverslips (22X22-1-002G; Epredia), and sealed with nail varnish. For the alternative staining protocol (Fig. 3), oocytes were washed three times in PBS (10 min each), stained with DAPI (1 μg/ml in PBS), and mounted in ProLong Gold Antifade Mountant (Invitrogen).

For REC8 visualization, chromosome spreads were prepared from metaphase I–arrested oocytes isolated from REC8-FKBP12^F36V^-mClover3 mice. Primary antibodies were applied overnight at 4°C, including anti-CENP-A (1:200; C51A7; Cell Signaling Technology) and anti-GFP (1:100; 11814460001; Roche). After washing, slides were incubated with secondary antibodies for 2 h at room temperature, including Alexa Fluor 488–conjugated anti-mouse (1:1,000; Molecular Probes) and Alexa Fluor 647–conjugated anti-rabbit (1:1,000; Molecular Probes).

### Targeted degradation of CENP-A by TRIM-Away

In CENP-A TRIM-Away experiments, prophase-arrested oocytes were coinjected with SNAP-TRIM21 mRNA and anti-CENP-A antibodies (10 pg per oocyte, rabbit monoclonal, Cell Signaling, C51A7, RRID:AB_1147629; or 140 pg per oocyte, custom rabbit polyclonal Ab1, Pacific Immunology, PAC-19283/19284) or control rabbit IgG (A-105-C; R&D Systems) in 0.05% (vol/vol) NP-40 in PBS. For partial CENP-A degradation, oocytes were injected with 2 pg of the commercial anti-CENP-A antibody (C51A7, 1:5 dilution). For rescue experiments, wild-type or CENP-B KO oocytes were coinjected with mRNA encoding mNeonGreen or mNeonGreen-CENP-C. After microinjection, oocytes were maintained in M2 medium supplemented with dbcAMP for 3 h to maintain prophase arrest, then transferred to M2 medium to resume meiosis. All TRIM-Away experiments and subsequent live imaging were conducted in M2 medium.

### Minor and major satellite FISH

After immunofluorescence imaging, oocyte slides were washed with PBS and treated with RNase A (1:200 dilution from a 20 mg/ml stock, 12091021; Invitrogen) in 2X SSC (EU0300-D; Euromedex) for 1 h at 37°C. Slides were washed twice in PBS and incubated with pepsin (P6887; Sigma-Aldrich; 0.125 mg/ml in 0.01 N HCl) for 10 min at 37°C. After PBS washes, slides were fixed in Carnoy’s solution (methanol:acetic acid, 3:1) and dehydrated in 80% ethanol for 1 min.

FISH was carried out using biotinylated probes that target either minor or major satellite sequences (diluted 1:100 from a 100 μM stock in hybridization buffer containing 10% dextran sulfate, 50% formamide, 2X SSC, and 1% Tween; see Table S2 for probe sequences synthesized at W.M. Keck Biotechnology Resource Lab at Yale University). The probe and sample were codenatured at 72°C for 2 min and then incubated overnight at 37°C in a humidified chamber. Slides were subsequently washed in 0.4X SSC at 72°C for 2 min, followed by washes in 2X SSC with 0.05% Tween-20 at room temperature for 30 s, and a final wash in PBS.

Slides were incubated with streptavidin-Alexa Fluor 647 (1:1,000 dilution from a 1 mg/ml stock, S21374; Invitrogen) in BlockAid blocking solution (B10710; Invitrogen), washed three times with PBS, and mounted for imaging.

### Sequential FISH and chromosome-specific identification

After removing the coverslip, oocyte slides were rinsed in 70% ethanol for 1 min. A prewarmed denaturation solution containing 70% formamide and 2X SSC at pH 7.0 was applied, and slides were incubated on a hotplate at 75°C for 2 min. The slides were then rinsed in 70% ethanol for 1 min and dehydrated sequentially in 90 and 100% ethanol for 1 min each. Samples were air-dried before hybridization with the probe mixture.

Chromosome-specific FISH staining was carried out following the manufacturer’s instructions (MetaSystems) using mouse whole chromosome painting probes (XMP probes, MetaSystems; see Table S3). In each experiment, two probes labeled with either FITC (green) or TRITC (orange) fluorochromes were used in a 1:1 ratio. Slides were mounted in ProLong Gold Antifade Reagent with DAPI (P36935; Invitrogen). Chromosome-specific FISH images were acquired using an upright Leica DM 6000B wide-field microscope equipped with a CoolSNAP HQ2 camera. Z-stacks up to 4 μm thick were collected at 1-μm intervals at room temperature utilizing a 40×HCX PL APO oil-immersion objective (NA 1.25). Acquisitions were done using MetaMorph software (Molecular Devices).

### Confocal live-cell imaging and immunofluorescence microscopy of oocytes and chromosome spreads

Confocal live imaging of oocytes was performed using Zeiss LSM 800 or Zeiss LSM 900 systems, each equipped with a 37°C environmental chamber and a 40×C-Apochromat water-immersion objective (NA 1.2). Z-stacks were captured using ZEN 2.6 at 6-min intervals, covering a total thickness of 70 μm with optical sectioning at 1.5–2.5 μm.

Confocal immunofluorescence images of intact oocytes and chromosomal spreads were acquired using ZEN 2.6 on Zeiss LSM 800 or Zeiss LSM 900 systems equipped with a 40×C-Apochromat water-immersion objective (NA 1.2). Fluorescence on Zeiss LSM 800 and 900 systems was detected using GaAsP/PMT/Airyscan detectors. For oocyte imaging, z-stacks were captured over a total thickness of 15 μm with a step size of 0.6 μm. For chromosomal spreads, z-stacks were collected over a total thickness of 4 μm with a step size of 0.3 μm. Control and experimental samples were imaged using the same acquisition settings. No nonlinear intensity transformations or γ adjustments were applied to any images. Cells were kept in M2 medium droplets under mineral oil during imaging.

For CD-1 meiosis I and meiosis II chromosome spreads (Fig. S2), imaging was conducted using a DeltaVision Core system (Applied Precision) mounted on an Olympus IX71 platform and equipped with a CoolSNAP HQ2 camera (Photometrics) and a 250 W xenon light source. Z-stacks up to 4 μm thick were captured at 0.2-μm intervals at room temperature, utilizing a 100×UPlanSApo oil-immersion objective (NA 1.4). The images were then deconvolved, and a 3D maximum-intensity projection was generated using SoftWoRx software.

For C57BL/6 chromosome spreads (Fig. 2), imaging was carried out using a Leica THUNDER Imager Live Cell system equipped with a Leica K8 CMOS back-thinned camera (95% quantum efficiency). Z-stacks up to 4 μm thick were collected at 0.2-μm intervals at room temperature using a 100×HCX PL APO oil-immersion objective (NA 1.4), followed by projection and postprocessing with Instant Computational Clearing in LAS X software.

### Measurement of fluorescence intensity in oocytes and chromosomal spreads

Fiji was used to quantify fluorescence intensity at centromeres in metaphase I oocytes and metaphase II eggs. Circular regions of interest of fixed size were manually placed over individual centromeres, and integrated density was measured in each channel on maximum-intensity projection images. Chromosome-specific images were used to assign centromeric signals to the corresponding chromosomes. For each spread, all visible centromeric signals were quantified and normalized to the mean centromeric intensity within that spread. A second normalization was performed by scaling signals from aged oocytes to the median centromeric intensity of young oocytes processed on the same day.

For the metaphase I chromosomal spreads, the ratio from the signal intensity of the “weakest” (h1) to the “strongest” (h2) homologous chromosome centromere was calculated and represented as a heatmap (Fig. S2 f). For intact live oocytes, the mean intensity of mNeonGreen-CENP-C fluorescence signal was obtained using the spot detection function in Imaris (Bitplane).

### 3D surface reconstruction and measurement of chromosome scattering volume

Chromosome surfaces were reconstructed in 3D from time-lapse movies of H2B-mScarlet using Imaris (Bitplane). Chromosome scattering volume was measured using object-oriented bounding box analysis, defined as the smallest cuboid enclosing all chromatids at each time point.

### Quantification of misaligned and lagging chromosomes during the first meiotic division and aneuploidy in chromosome spread of the second meiotic division

Chromosomes were classified as misaligned if they did not align at the metaphase plate in the frame immediately before anaphase onset, based on previously defined criteria (Mogessie and Schuh, 2017). Lagging chromosomes were defined as those remaining between segregating chromosome masses 18 min after anaphase onset (three frames under the acquisition settings used). Both misaligned and lagging chromosomes were manually identified from time-lapse movies and validated using 3D isosurface reconstructions generated for chromosome scattering analysis.

Aneuploidy frequency was quantified in metaphase II chromosome spreads from young and aged eggs. To avoid overestimating defects, we excluded overspread cells, defined as those with chromosomes extending beyond the field of view (134 × 134 μm; see microscopy methods), as this could compromise accurate chromosome counts per oocyte.

### Sample preparation for electron tomography

Metaphase II–stage mouse oocytes were collected in cellulose capillary tubes (Pelletier et al., 2006) and high-pressure–frozen as described using an EM ICE high-pressure freezer (Leica Microsystems) (Müller-Reichert et al., 2007). Freeze substitution was performed over 2–3 days at −90°C in anhydrous acetone containing 1% OsO_4_, 0.1% uranyl acetate, and 2% H_2_O. Samples were embedded in Epon/Araldite and polymerized for 2 days at 60°C.

Serial semi-thick sections (200 nm) were cut using a Leica Ultracut S microtome (Leica Microsystems), collected on Pioloform-coated copper slot grids, and poststained with 2% uranyl acetate in 70% methanol followed by Reynolds’ lead citrate. For dual-axis electron tomography, 15-nm colloidal gold particles (Sigma-Aldrich) were attached to both sides of semi-thick sections to serve as fiducial markers for subsequent image alignment. A series of tilted views was recorded using an F20 electron microscope (Thermo Fisher Scientific, formerly FEI) operating at 200 kV using a Gatan US4000 (4,000 px×4,000 px) CCD camera. Images were captured every 1.0° over a ±60° range.

### Quantification of electron tomography data

We used the IMOD software package (http://bio3d.colorado.edu/imod) for calculating electron tomograms (Kremer et al., 1996). We performed initial segmentation and automatic tracing of microtubules in the tomographic volumes using the newly developed TARDIS (Kiewisz et al., 2024, *Preprint*). We used an extension to the filament editor of the Amira visualization and data analysis software (Redemann et al., 2014; Stalling et al., 2005; Weber et al., 2012). We also used Amira to stitch the obtained 3D models in *Z* to create complete volumes of the recorded spindles (Lindow et al., 2021). The automatic segmentation of the spindle microtubules was followed by a visual inspection of the traced microtubules within the tomograms. Correction of individual microtubule tracings involved manually tracing undetected microtubules, connecting microtubules across sections, and removing tracing artifacts (e.g., vesicle membranes). We generated and rendered movies of tomographic reconstructions using the freely available Blender software.

### Data analysis for tomography

Data analysis was performed using Amira software (Visualization Sciences Group).

### Classification of microtubules

For the detection of possible microtubule and chromosome interactions in 3D, all microtubules that reached 200 nm of the chromosome surface were detected using the *SpatialGraphDistanceToSurface* module in Amira. This tool measures the nearest distance of each microtubule lattice and end to the segmented chromosome surface. Using the *Filter* module, we selected microtubules based on their distance to the surface. For our quantification, we selected any microtubule that reached within 200 nm (Redemann et al., 2017). We repeated the analysis using distance thresholds from 100 to 400 nm; changing the threshold did not alter the descriptive patterns. To distinguish between end-on and laterally attaching microtubules to the chromosome surface, we used the *EndExtrapolation* module in the *SpindleAnalys* Toolbox. This tool quantifies microtubule attachment to chromosomes and distinguishes end-on from lateral attachments based on the angle between the microtubule and the chromosome surface.

### Microtubule interaction analysis

To detect possible interactions in 3D, the *Interaction* module of the *SpindleAnalysis* Toolbox was used in Amira. Interaction computes possible microtubule intersections. An intersection is defined as a part of a microtubule that is closer to another microtubule than a given threshold (here, 100 nm). In detail, for each point of a microtubule, the distance to another microtubule is computed. Based on these distances, we detect all parts of the microtubule that lie closer to the other microtubule than the threshold. For each part, we generate an interaction and store it in a spreadsheet. For each interaction, we score the approximated angle and the interaction start, end, and length. In addition, we defined a minimal interaction length of 25 nm, over which the distance between the interacting microtubules must remain within the defined threshold distance for an interaction (here, 100 nm) to filter the interactions.

The distance module computes the distance between microtubules. For each point of a microtubule, the closest segment of all other microtubules is detected and stored as a floating-point attribute. Furthermore, for each microtubule, the closest other microtubule is detected. The distance between two microtubules is defined as the minimal distance between the segments. The ID and distance are stored as edge attributes.

### Microtubule orientation analysis

To evaluate the degree of disorganization of microtubules interacting with chromosomes in meiotic spindles, the *Orientation by Axis* module of the *SpindleAnalysis* Toolbox in Amira was employed. This tool calculates the angles of individual microtubules relative to a reference axis defined by two input points. The direction of each microtubule is approximated using a vector connecting its start and end points, and the orientation is quantified as the angle between this vector and the spindle axis vector. The direction is inverted if it points opposite the reference axis, limiting the angles to 0 to π/2. This approach ensures consistent measurement of microtubule alignment within the spindle.

### Statistical analysis

All statistical analyses were performed using GraphPad Prism. Box plots display the median (center line), 25th and 75th percentiles (interquartile range), and the minimum and maximum values (whiskers). Data were normalized by dividing each value in both the control and experimental groups by the control group mean. Alternative normalization methods are detailed in the corresponding figure legends. First, we tested each dataset for normality using the D’Agostino and Pearson test. If one or more datasets failed the test, we used nonparametric data analyses. The tests used for each dataset are described in the respective figure legend. For chromosome scatter volume analysis, the timing from GVBD to anaphase was not constant, leading to missing values. Because ANOVA cannot accommodate missing data, we used a mixed-effects model to analyze these data. When there were no missing values, the mixed-effects model produced identical P values to repeated-measures ANOVA; with missing values, it provided a valid alternative with the same interpretation. Statistical tests are specified in the figure legends. Significance thresholds were set at *P < 0.05, **P < 0.01, ***P < 0.001, and ****P < 0.0001. See Table S4 for source dataset.

### Online supplemental material

Fig. S1 shows ultrastructural and quantitative analysis of metaphase II spindles in young and aged eggs. Fig. S2 shows age-associated changes in centromere composition in oocytes and eggs. Fig. S3 shows CENP-A TRIM-Away phenotypes are reproducible using an independent custom antibody. Fig. S4 shows effects of CENP-A depletion on centromeric REC8 and CENP-C recruitment and meiotic progression in control and CENP-B KO oocytes. Fig. S5 shows partial CENP-A depletion induces mild chromosomal abnormalities during meiosis I. Video 1 shows 3D tomographic reconstruction of cell 1. Video 2 shows 3D tomographic reconstruction of cell 4. Video 3 shows CENP-A TRIM-Away using CENP-A Ab2–Control. Video 4 shows CENP-A TRIM-Away using CENP-A Ab2–CENP-A Ab2. Video 5 shows CENP-A TRIM-Away using CENP-A Ab1–Control. Video 6 shows CENP-A TRIM-Away using CENP-A Ab1–CENP-A. Video 7 shows exogenous expression of CENP-C in CENP-A TRIM-Away oocytes–Control + mNeonGreen. Video 8 shows exogenous expression of CENP-C in CENP-A TRIM-Away oocytes–Control + mNeonGreen-CENP-C. Video 9 shows exogenous expression of CENP-C in CENP-A TRIM-Away oocytes–CENP-A + mNeonGreen. Video 10 shows exogenous expression of CENP-C in CENP-A TRIM-Away oocytes–CENP-A + mNeonGreen-CENP-C. Video 11 shows exogenous expression of CENP-C in CENP-B KO oocytes–Control + mNeonGreen. Video 12 shows exogenous expression of CENP-C in CENP-B KO oocytes–Control + mNeonGreen-CENP-C. Video 13 shows exogenous expression of CENP-C in CENP-B KO oocytes–CENP-A TRIM-Away + mNeonGreen. Video 14 shows exogenous expression of CENP-C in CENP-B KO oocytes–CENP-A TRIM-Away + mNeonGreen-CENP-C. Video 15 shows partial CENP-A TRIM-Away using CENP-A Ab2–Control. Video 16 shows partial CENP-A TRIM-Away using CENP-A Ab2–CENP-A Ab2. Video 17 shows exogenous expression of CENP-C in aged oocytes–Young + mNeonGreen. Video 18 shows exogenous expression of CENP-C in aged oocytes–Aged + mNeonGreen. Video 19 shows exogenous expression of CENP-C in aged oocytes–Aged + mNeonGreen-CENP-C. Table S1 shows oligonucleotide sequences used in this study (shown 5′-3′). Table S2 shows minor (covering 121 bp) and major satellite sequences (covering 234 bp) used in the study. Table S3 shows list of mouse chromosome painting probes (MetaSystems Probes) used for whole chromosome FISH in meiosis II chromosome spreads. Table S4 shows source data.

## Supplementary Material

Table S1 shows oligonucleotide sequences used in this study (shown 5′-3′).

Table S2 shows minor (covering 121 bp) and major satellite sequences (covering 234 bp) used in the study.

Table S3 shows list of mouse chromosome painting probes (MetaSystems Probes) used for whole chromosome FISH in meiosis II chromosome spreads.

Table S4 shows source data.

## Data Availability

The data underlying Figs. 1, 2, 3, 4, 5, and 6 and Figs. S1, S2, S3, S4, and S5 are available in the published article and its online supplemental material.
