# Supplementary material for: Declining centromere strength compromises meiotic chromosome segregation in aging oocytes
Source: J Cell Biol. 2026 Oct 19;225(11):e202606132. doi: 10.1083/jcb.202606132 (PMC13679647; doi:10.1083/jcb.202606132)
Supplement: Supplementary file 1 [file jcb_202606132_tables1.docx]

**Table S1. Oligonucleotide sequences used in this study (shown 5’-3’)**

| Primer Name | Primer Sequence |
| --- | --- |
| KS109 | AGCGCTACCGGTCTCAGATCATGCCAGAGCCAGCGAAG |
| KS110 | TCCCGGGCCGTCGACTGCAGAATTCTTAGCGCTGGTGTACTTGG |
| KS111 | GGTCGCCACCATGGTGAGCAAGGGCGAG |
| KS112 | ACTCCTTAAGCTTGTACAGCTCGTCCATGC |
| TL12 | CCTGCAGGCGGATCCGCG |
| TL13 | GGTGAATTCACCGGTAGCGCTAGC |
| TL14 | GCGCTACCGGTGAATTCACCATGGTGAGCAAGGGCGAG |
| TL15 | AACGCGGATCCGCCTGCAGGCTTGTACAGCTCGTCCATGC |
| BM489 | ATGATAGCTTGATCTGGTTACC |
| BM490 | CTTGTACAGGATCCCGGG |
| BM491 | ggcccgggatcctgtacaagCCGGCCGGTCGCCACCATGGTGAGCAAG |
| BM492 | taaccagatcaagctatcatTTACTTGTACAGCTCGTCCATGCCGC |
| BM729 | TCGATGCCCTTCAGCTCGAT |
| BM730 | CCACCCTCGTGACCACCT |
| BM732 | GGCTGCTACAGCACACTCTA |
| BM733 | CCACCCCGGTGAACAGCT |
| BM734 | GTTATCCCAGCTGACACCCC |
| BM735 | AAAGGTGGCCTGTCTTCTGC |
| BM764 | TAGATAAATGATAGCTTGATCTGGTTACC |
| BM765 | AGCGGCCGCTTTACTTGTAC |
| BM766 | GTACAAGTAAAGCGGCCGCTATGGTGAGCAAGGGCGAG |
| BM767 | ATCAAGCTATCATTTATCTATTACTTGTACAGCTCGTCCATG |
| BM830 | CAGCTGACGTTCCGGGAGAA |
| BM831 | GGGGACAGCTTGTTGGTCTT |
