# Supplementary material for: Declining centromere strength compromises meiotic chromosome segregation in aging oocytes
Source: J Cell Biol. 2026 Oct 19;225(11):e202606132. doi: 10.1083/jcb.202606132 (PMC13679647; doi:10.1083/jcb.202606132)
Supplement: Supplementary file 2 [file jcb_202606132_tables2.docx]

**Table S2: Minor (covering 121 bp) and major satellite sequences (covering 234 bp) used in the study.** An equimolar mixture of two (minor satellite) and six (major satellite) single-strand biotinylated oligos was used for the FISH staining.

| minor satellite | 1 | GGAAAAUGAUAAAAACCACACUGUAGAACAUAUUAGAUGAGUGAGUUACACUGAAAAACA |
| --- | --- | --- |
|  | 2 | CAUUCGUUGGAAACGGGAUUUGUAGAACAGUGUAUAUCAAUGAGUUACAAUGAGAAACAU |
| major satellite | 1 | DSSGGACCUGGAAUAUGGCGAGAAAACUGAAAAUCACGGAAAAUGAGAAAUACACACUUUAGGa |
|  | 2 | DSSUCACGGAAAAUGAGAAAUACACACUUUAGGACGUGAAAUAUGGCGAGGAAAACUGAAAAa |
|  | 3 | DSSACGUGAAAUAUGGCGAGGAAAACUGAAAAAGGUGGAAAAUUUAGAAAUGUCCACUGUAGg |
|  | 4 | DSSGGUGGAAAAUUUAGAAAUGUCCACUGUAGGACGUGGAAUAUGGCAAGAAAACUGAAAAUc |
|  | 5 | DSSACGUGGAAUAUGGCAAGAAAACUGAAAAUCAUGGAAAAUGAGAAACAUCCACUUGACGAc |
|  | 6 | DSSAUGGAAAAUGAGAAACAUCCACUUGACGACUUGAAAAUGACGAAAUCACUAAAAAACGUg |
