# Supplementary material for: Declining centromere strength compromises meiotic chromosome segregation in aging oocytes
Source: J Cell Biol. 2026 Oct 19;225(11):e202606132. doi: 10.1083/jcb.202606132 (PMC13679647; doi:10.1083/jcb.202606132)
Supplement: Supplementary file 3 [file jcb_202606132_tables3.docx]

**Table S3: List of mouse chromosome painting probes (Metasystems probes) used for whole chromosome FISH in meiosis II chromosome spreads.** Two probes, directly labeled with a green or orange-emitting fluorochrome, were used in a 1:1 mix for each experiment.

| D-1401-050 | XMP 1 Mouse Chromosome 1 Painting Probe |
| --- | --- |
| D-1402-050 | XMP 2 Mouse Chromosome 1 Painting Probe |
| D-1403-050 | XMP 3 Mouse Chromosome 3 Painting Probe |
| D-1404-050 | XMP 4 Mouse Chromosome 4 Painting Probe |
| D-1405-050 | XMP 5 Mouse Chromosome 5 Painting Probe |
| D-1406-050 | XMP 6 Mouse Chromosome 6 Painting Probe |
| D-1407-050 | XMP 7 Mouse Chromosome 7 Painting Probe |
| D-1408-050 | XMP 8 Mouse Chromosome 8 Painting Probe |
| D-1409-050 | XMP 9 Mouse Chromosome 9 Painting Probe |
| D-1410-050 | XMP 10 Mouse Chromosome 10 Painting Probe |
| D-1411-050 | XMP 11 Mouse Chromosome 11 Painting Probe |
| D-1412-050 | XMP 12 Mouse Chromosome 12 Painting Probe |
| D-1413-050 | XMP 13 Mouse Chromosome 13 Painting Probe |
| D-1415-050 | XMP 15 Mouse Chromosome 15 Painting Probe |
| D-1416-050 | XMP 16 Mouse Chromosome 16 Painting Probe |
| D-1417-050 | XMP 17 Mouse Chromosome 17 Painting Probe |
| D-1418-050 | XMP 18 Mouse Chromosome 18 Painting Probe |
| D-1419-050 | XMP 19 Mouse Chromosome 19 Painting Probe |
| D-1420-050 | XMP X Mouse Chromosome X Painting Probe |
